# The Bacteriophage Pf-10—A Component of the Biopesticide “Multiphage” Used to Control Agricultural Crop Diseases Caused by *Pseudomonas syringae*

**DOI:** 10.3390/v14010042

**Published:** 2021-12-27

**Authors:** Olesya A. Kazantseva, Rustam M. Buzikov, Tatsiana A. Pilipchuk, Leonid N. Valentovich, Andrey N. Kazantsev, Emilia I. Kalamiyets, Andrey M. Shadrin

**Affiliations:** 1 Laboratory of Bacteriophage Biology, G. K. Skryabin Institute of Biochemistry and Physiology of Microorganisms, Pushchino Scientific Center for Biological Research of the Russian Academy of Sciences, Federal Research Center, 142290 Pushchino, Russia; a87h5n1@gmail.com; 2 Institute of Microbiology, The National Academy of Sciences of Belarus, 220141 Minsk, Belarus; tanya.pilipchuk@tut.by (T.A.P.); valentovich@mbio.bas-net.by (L.N.V.); kolomiets@mbio.bas-net.by (E.I.K.); 3 Faculty of Biology, Belarusian State University, 220030 Minsk, Belarus; 4 P. N. Lebedev Physical Institute of the Russian Academy of Sciences, Pushchino Radio Astronomy Observatory, 142290 Pushchino, Russia; kaz.prao@bk.ru

**Keywords:** *Pseudomonas syringae*, phytopathogenic bacteria, bacteriophage, phage, biopesticide

## Abstract

Phytopathogenic pseudomonads are widespread in the world and cause a wide range of plant diseases. In this work, we describe the *Pseudomonas* phage Pf-10, which is a part of the biopesticide “Multiphage” used for bacterial diseases of agricultural crops caused by *Pseudomonas syringae*. The Pf-10 chromosome is a dsDNA molecule with two direct terminal repeats (DTRs). The phage genomic DNA is 39,424 bp long with a GC-content of 56.5%. The Pf-10 phage uses a packaging mechanism based on T7-like short DTRs, and the length of each terminal repeat is 257 bp. Electron microscopic analysis has shown that phage Pf-10 has the podovirus morphotype. Phage Pf-10 is highly stable at pH values from 5 to 10 and temperatures from 4 to 60 °C and has a lytic activity against *Pseudomonas* strains. Phage Pf-10 is characterized by fast adsorption rate (80% of virions attach to the host cells in 10 min), but has a relatively small number of progeny (37 ± 8.5 phage particles per infected cell). According to the phylogenetic analysis, phage Pf-10 can be classified as a new phage species belonging to the genus *Pifdecavirus*, subfamily *Studiervirinae*, family *Autographiviridae*, order *Caudovirales*.

## 1. Introduction

*Pseudomonas* is a genus of Gram-negative bacteria including, *inter alia*, the known plant pathogen species. The phytopathogenic pseudomonads cause a wide range of plant diseases: necrotic lesions of fruit [1,2], stems [1,3], and leaves [1,2,4], hyperplasias (galls) [5], tissue macerations (rots) [6,7], cankers [1,8,9], blights [10], and vascular infections (wilts) [11]. Plant diseases caused by *Pseudomonas* are widespread throughout the world and infect a large number of higher plants, including agriculturally important crops, resulting in the loss of agribusiness profits and other consequential damages that ultimately have a negative impact on the world food supplies and economy. Moreover, the phytopathogen-infested plants can be a source of toxic molecules [12,13].

*Pseudomonas syringae* is one of the most widespread and best studied *Pseudomonas* plant pathogens. Depending on the range of hosts and the type of symptoms they cause, these pathogens are assigned to different intraspecific taxa called pathovars [14]. Within *Pseudomonas syringae*, there are at least 60 pathovars [15]. In particular, *Pseudomonas syringae* pv. *tomato* is the causal agent of tomato speck disease [16]; *P. syringae* pv. *syringae* cause blossom blast of apple [17] and pear [2], apical necrosis of mango [18], canker of cherry [19] and plum [20] and brown spot of beans [21]; *Pseudomonas syringae* pv. *atrofaciens* cause brown bacteriosis of grain crops [22,23]; etc. The characteristic symptoms of *Pseudomonas syringae* damage to plants are chlorosis and necrosis of tissue partially caused by toxins such as coronatine [16,24], syringomycin [4], syringopeptin [25] and antimetabolite toxins (tabtoxin, phaseolotoxin and mangotoxin) [12]. It should also be noted that strains have been found, which, although phylogenetically belong to *Pseudomonas syringae*, are not phytopathogens and exist as commensals on plants [26].

An essential part of the agricultural sector is the protection of crops from pathogens at all stages of plant growth. The currently used agriculture strategies for controlling bacterial infections (e.g., bactericide applications such as copper compounds [27,28,29], antibiotic applications [30], etc.) can pose a serious threat to both environment and public health. Moreover, the situation is aggravated by the emergence of antimicrobial-resistant bacteria, including *Pseudomonas*. A potential alternative to the traditional measures of bacterial disease control is the bacteriophage-based biocontrol [31]. Bacteriophages or phages are the natural enemies of bacteria and can be successfully used as therapeutic or prophylactic agents against bacteria-related plant diseases [32]. Currently, new phages have been isolated and characterized for different pathovars of *Pseudomonas syringae* [32,33,34,35,36,37,38].

In this work, we have characterized *Pseudomonas* phage Pf-10, which is a component of the biopesticide “Multiphage” [39] used for bacterial diseases of agricultural crops caused by *Pseudomonas syringae*. According to the phylogenetic analysis, phage Pf-10 is a new phage species belonging to the genus *Pifdecavirus* (subfamily *Studiervirinae*, family *Autographiviridae*, order *Caudovirales*). The article presents the detailed data on phage morphology, stability, genome organization, phylogenetic relationship and parameters of phage infection (adsorption, one-step growth curve) to provide biological insights of phages that can be used as the agents for controlling plant diseases caused by *Pseudomonas*.

## 2. Materials and Methods

### 2.1. Bacterial Strains and Culture Conditions

The bacterial strains used in this study were obtained from the Belarusian collection of non-pathogenic microorganisms (BIM) of the Institute of Microbiology, National Academy of Sciences, Minsk, Belarus. All strains were cultivated on 1.5% LB agar plates [10.0 g/L tryptone; 5.0 g/L yeast extract; 10.0 g/L sodium chloride, 15.0 g/L bacto agar] at 28 °C and used for preparation of bacterial cultures. The bacterial cultures were grown in lysogeny broth (LB) at 28 °C with shaking at 120 rpm.

### 2.2. Phage Isolation and Propagation

Phage Pf-10 was isolated from a tissue sample of green bean (*Phaseolus vulgaris*) infected with *P. syringae* pv. *syringae* in 2014 [40]. Briefly, the plant material was collected in the Vitebsk Oblast, Belarus. The sensitive strain *P. fluorescens* BIM B-582 was used as the host strain for initial propagation. *P. fluorescens* BIM B-582 culture was grown in GRM broth [8.0 g/L pancreatic hydrolysate of fish-meal, 8.0 g/L enzymatic peptone, 4.0 g/L NaCl] (Obolensk, Russia) at 28 °C with shaking (250 rpm) overnight. The culture was then diluted 10-fold in a fresh medium and incubated at 28 °C with shaking (250 rpm) to OD_590_ of 0.8. The plant sample was pounded in a mortar with 30 mL of GRM broth, followed by centrifugation at 5000× *g* for 30 min. Then, 25 mL of the supernatant was mixed with 25 mL of a BIM B-582 culture and incubated for 18 h at 28 °C with shaking (250 rpm). The cell debris was removed by centrifugation at 6000× *g* for 30 min, and the supernatant was analyzed for the presence of phages. Phage assay was carried out by using the double agar layer method. After overnight incubation at 28 °C, the plates were examined for plaques. A separate plaque was selected and used for further phage propagation, and the resultant phage preparation was used to obtain separate plaques. The propagation-plaque selection cycle was repeated five times in order to exclude the presence of other phages. Phage Pf-10 was deposited into the Belarusian collection of non-pathogenic microorganisms (BIM) of the Institute of Microbiology, National Academy of Sciences, Minsk, Belarus under the collection number BIM BV-61-D [39].

The sensitive strain *P. syringae* BIM B-268 [41] was used for phage propagation to obtain high-titer suspension. Briefly, 500 μL of the overnight bacterial culture was transferred into 50 mL of LB with 10 mM CaCl_2_ and 10 mM MgCl_2_ and incubated at 28 °C with shaking at 120 rpm to the OD_590_ of 0.2 (at approximately 1 × 10^8^ colony-forming units (CFU)/mL). Then, the 25 μL phage sample (2 × 10^8^ plaque-forming units (PFU)/mL) was mixed with 50 mL of the BIM B-268 culture (OD_590_ of 0.2) with a multiplicity of infection (MOI) of 0.001 and incubated at 28 °C with shaking at 120 rpm for 6 h until the complete lysis of the bacterial culture. After the lysis, PEG 8000 (polyethylene glycol 8000) precipitation was performed as described previously [42]. The PEG-precipitated phage sample was dissolved in SM buffer [50 mM Tris-HCl, pH 7.5; 100 mM NaCl; 1 mM MgSO_4_; 0.01% gelatin]. The resultant phage preparation was filtered through a 0.22 μm sterile filter and stored at 4 °C. Phage titer was determined by the double agar overlay plaque assay method. The Pf-10 phage titer in the final high-titer preparation was 5 × 10^11^ PFU/mL.

### 2.3. Transmission Electron Microscopy

Three mL of high-titer phage preparation were centrifuged using the preformed CsCl gradient (1.25 g/mL, 1.4 g/mL, 1.5 g/mL and 1.7 g/mL, 2.5 mL each) at 10 °C, 25,000 rpm, for 2.5 h in a Beckman Coulter ultracentrifuge, L7-55, using a SW 41 Ti rotor. Further, ten µL of the concentrated phage suspension (10^9^ PFU/mL) were applied to carbon-coated copper grids (400 mesh) and negatively stained with 1% uranyl acetate. The grids were analyzed using a JEM 1200EX (JEOL, Tokyo Japan) transmission electron microscope at 80 kV accelerating voltage. Images were taken on Kodak film SO-163 (Kodak, Cat. \# 74144, Hatfield, PA, USA). Phage particle dimensions were measured using ImageJ version 1.53e in relation to the scale bar generated by the microscope.

### 2.4. Host Range

Phage host range analysis was performed by spot test using 45 strains of the genus *Pseudomonas* (Supplementary Information, Table S1). Five μL of the phage suspension (10^9^ PFU/mL) were dripped onto agar plates with the different strains, followed by incubation at 28 °C for 20 h.

### 2.5. Thermal and pH Stability Tests

Phage stability tests were conducted as described previously [43], with some modifications. Briefly, the phage stability at various temperatures (4, 30, 40, 50, 60, 70, 80 and 90 °C) was investigated by incubating the phage (at approximately 5 × 10^9^ PFU/mL) at the respective temperatures for 1 h. Phage stability at various pH values (ranging from 2.2 to 10) was evaluated using four buffers: Glycine-HCl buffer (pH values 2.2 and 3), sodium acetate buffer (pH 4 and 5), phosphate buffer (pH 6, 7 and 8) and Glycine-NaOH buffer (pH 9 and 10). The phage sample was mixed with each buffer to a final phage concentration of 5 × 10^9^ PFU/mL and incubated at 28 °C for 1 h. After incubation, the phage titer was determined by double-layer plate titration. Three independent trials of the experiment were performed. The results were processed using GraphPad Prism 8.4.3 as the mean of three observations ± standard deviation.

### 2.6. The Effects of Ca^2+^ and Mg^2+^ on the Killing Activity of Pf-10

In order to assess the killing activity of Pf-10 and the calcium and magnesium effects on this process, the BIM B-268 culture was infected with the phage at MOI values of 10, 1 and 0.1 in the presence or absence of Ca^2+^ and/or Mg^2+^ ions in solution. Briefly, 50 µL of phage preparations (at approximately 8 × 10^9^, 8 × 10^8^, 8 × 10^7^ PFU/mL) were mixed separately with 450 µL of the log-phase BIM B-268 culture (at approximately 8 × 10^7^ CFU/mL) in a 48-well microplate. A non-infected BIM B-268 culture was used as a control to assess the killing activity. In order to estimate the effects of Ca^2+^ and/or Mg^2+^ ions, CaCl_2_ and/or MgCl_2_ were added to the phage-host mixtures (to the final concentration of 10 mM each), SM buffer [50 mM Tris-HCl, pH 7.5; 100 mM NaCl; 1 mM MgSO_4_; 0.01% gelatin] was added as a control. Then the microplate was incubated at 28 °C for 3 h in a FilterMax F5 microplate reader (Molecular Devices, San Jose, CA, USA) with the following shaking settings: shake mode—orbital, shake intensity—medium, with OD_595_ being measured every 10 min. Three independent trials of the experiment were performed. The results were reported as the mean of three observations ± standard deviation. The resultant growth curves were visualized using GraphPad Prism 8.4.3.

The Pf-10 lytic activity in the presence or absence of Ca^2+^ and/or Mg^2+^ ions was evaluated using the PhageScore method developed by Konopacki [44]. Briefly, a fitting of the data on the growth kinetic of *P. syringae* BIM B-268 upon Pf-10 infection at different MOI was performed by non-linear least-squares method using the LMFIT library (https://lmfit.github.io/lmfit-py/intro.html, accessed 29 September 2021). To fit the growth curves of the control (non-infected BIM B-268 culture) and test (the BIM B-268 culture infected by the Pf-10 phage) samples, formulas 1 and 2 described by Konopacki [44] were used, respectively. To calculate the PhageScore values (P_S_) of the Pf-10 phage at different MOI (P_Si_, PhageScore for given MOI_i_, i—the number of different MOI values for the same bacteriophage), we used the area ratios obtained by the mathematical fitting of functional dependencies, formula 8 in Konopacki’s article [44]. The areas under the curve were calculated using Simpson’s rule. The value of the Phage Total Score factor (P_TS_) was calculated using formula 12 from the article [44].

### 2.7. Adsorption Assay

In order to determine the attachment time of the phage to a bacterial cell, an adsorption assay was performed according to the protocol developed by Kropinski [45]. Briefly, Eppendorf tubes containing 0.95 mL of LB broth with three drops of chloroform were placed on ice to chill for 10 min. Nine mL of the log-phase BIM B-268 culture (OD_590_ of 0.2, approximately 1 × 10^8^ CFU/mL) were transferred into a 100-mL laboratory flask and incubated in a shaking water bath at 28 °C, 60 rpm for 5 min. Nine mL of LB broth were used as a control. Further, 1 mL of the phage suspension, preheated for 5 min at 28 °C, with a concentration of 1 × 10^7^ PFU/mL was added into the test flask to provide MOI values of 0.01. The phage suspension was added into the control flask in the similar way. Then 50-µL aliquots were taken from both test and control flasks every minute, transferred into the prepared Eppendorf tubes and vortexed vigorously. The obtained mixtures were serially diluted in SM buffer and quantified using double agar overlay plaque assay. Five independent trials of the experiment were performed. The results were presented as percentages of the initial phage number and processed using GraphPad Prism 8.4.3 with error bars representing standard deviation for five trials. The adsorption rate was calculated using the equation described by Kropinski [45].

### 2.8. One-Step Growth Curve

In order to determine the average burst size of the phage, a one-step growth experiment was carried out as described by Hyman and Abedon [46]. One mL of phage suspension (1 × 10^7^ PFU/mL), preheated for 5 min at 28 °C, was added to 9 mL of the bacterial culture (1 × 10^8^ CFU/mL) to provide MOI values of 0.01. The resultant mixture was incubated in a shaking water bath at 28 °C, 60 rpm for 6 min for phage adsorption. After that, a 1-mL aliquot was transferred into a 1.5 mL Eppendorf tube and centrifuged at 3500× *g* for 10 min at 4 °C to precipitate the cells. The pellet was resuspended in 1 mL of fresh LB broth followed by addition to 9 mL of LB into a 100-mL laboratory flask and incubation in a shaking water bath at 28 °C, 60 rpm. The 50-µL aliquots of the mixture were collected at 10-min intervals for 1 h and diluted in SM buffer followed by phage quantification using double agar overlay plaque assay. Three independent trials of the experiment were performed. The PFU/mL values were calculated and plotted against time. The results were processed using GraphPad Prism 8.4.3 with error bars representing standard deviation for three trials. The latent period was determined as the interval between the adsorption of the phages to the bacterial cells and the release of phage progeny. The burst size of the phage Pf-10 was determined as the ratio of the average number of free phage particles after the release phase (plateau average [PFU/mL]) to their number during the latency phase (latent average [PFU/mL]).

### 2.9. Genome Sequencing, Assembly and Sequence Analysis

Phage preparation was treated with DNase I and RNase A. Phage DNA was extracted using DNeasy Blood & Tissue Kit (Qiagen, Hilden, Germany) according to the manufacturer’s instructions. The library for genome sequencing was constructed using a Nextera XT DNA Library Prep Kit (Illumina Inc., San Diego, CA, USA) and sequenced on the Illumina MiSeq platform using a MiSeq reagent kit v.3 (2 × 300 bp). The genome assembly was performed by SPAdes v. 3.1 [47], and resulted in a single contig with the average coverage of 788×. Open reading frames (ORFs) were identified using RASTtk v.2.0 [48] followed by functional annotation using BLASTp v2.13.0+ (NCBI) [49] and HHpred (accessed 12 May 2021) [50]. ARAGORN v1.2.41 [51] was used to identify putative tRNAs and transfer-messenger RNAs. BRIG software v. 0.95 [52] was used for genome visualization. Phage protein were compared using the PSI-BLAST (Position-Specific Iterated BLAST) algorithm [49].

### 2.10. The Genome Packaging Strategy

Standard restriction analysis was used to confirm the correctness of genome assembly and identification of the bacteriophage genome termini of Pf-10 as described previously [43]. In order to determine the ends of the Pf-10 genome more accurately, rapid amplification of the ends of the genome (RAGE) was performed, as described previously [43], with minor changes to the protocol. Phage DNA was used for a typical DNA tailing reaction with the terminal transferase (New England Biolabs, Ipswich MA, USA, Cat. # M0315L) according to the protocol provided by the enzyme manufacturer. Further, two PCRs were carried out sequentially using the TaqSE DNA polymerase (SibEnzyme, Novosibirsk, Russia, Cat. # E314) and the pairs of oligonucleotides designed for the right and left ends of the Pf-10 genome (Supplementary Information, Table S3). The 3’-end tailing fragments were used as the DNA template for the first PCR, and the product of the first PCR was used as the template for the second reaction. The final PCR products were extracted from electrophoresis gel and used for Sanger sequencing with primers Pf-10_R_for2 5′-CCTTTAGGGTGCAGCACATC-3′ and Pf-10_L_rev2 5′-TGATGGTCCTCTATGGGCCT-3′ for the right and left ends of the genome, respectively. Due to the limitations of the RAGE method in locating the genome ends in the sequences containing “A” nucleotides, the right end of the Pf-10 genome was re-sequenced using the genomic DNA of phage Pf-10 as a template. The additional Sanger sequencing reaction for the right genome end was performed using a LiCor4300 DNA analyzer (LI-COR, Lincoln, NE, USA) with the primer Pf-10-t 5′-TGAGAATCATGTGCTATCTG-3′.

The genome packaging strategy of Pf-10 was predicted by phylogenetic analysis of the large terminase subunit as described by Merrill [53]. 57 large terminase proteins were aligned using MAFFT v7.308 [54] with default settings. The phylogenetic tree was computed using the neighbor-joining method by MEGA X [55] with a bootstrapping set to 100 and was visualized using FigTree v1.4.4 [56] with midpoint rooting.

### 2.11. Comparative Genomics

In order to find the related phages, ViPTree server version 1.9 [57] was used to generate a proteomic tree based on the genome-wide sequence similarities computed by tBLASTx. In addition to the reference virus genomes from the ViPTree server database, 10 phage genomes found by the BLASTn search using the whole Pf-10 genome sequence as the query were added to create the proteomic tree (Supplementary Information, Table S5). A linear comparison diagram showing the genomic identity between Pf-10 and the most closely related phages was also created with the ViPTree server version 1.9 [57]. The number of shared proteins was computed using the GET_HOMOLOGUES software v3.3.3 [58] with the COGtriangles algorithm [59] (−t 0 −C 75 −e).

### 2.12. Accession Number

The phage genome sequence was deposited into the GenBank database under the accession number KP025626 and NCBI Reference Sequence database under the accession number NC_027292.1 (BioProject accession number PRJNA263998, BioSample accession number SAMN21529907). The raw sequence dataset is available at the Sequence Read Archive under the accession number SRR15990561.

### 2.13. Statistical Analysis

All data comparisons performed in this study were verified using statistical analyses in order to ascertain the significance of the results. The statistical analyses were performed using GraphPad Prism 8.4.3. One-way ANOVA with repeated measures was used to assess the significance of differences in phage concentrations between control and tested samples during phage stability experiments (Section 3.2. *pH and Thermal Stability Tests*). Two-way ANOVA with repeated measures was used to analyze the statistical differences between the growth curves of the non-infected and infected *P. syringae* BIM B-268 cultures upon Pf-10 infections at different MOI (Section 3.3. *Killing Assay and Effect of Ca^2+^ and Mg^2+^ on the Killing Activity of Pf-10*). Two-way ANOVA with repeated measures was also used to analyze the statistical differences between the growth curves of the *P. syringae* BIM B-268 upon Pf-10 infections at different MOI in the presence and absence of *Ca^2+^ and Mg^2+^* ions (Section 3.3. *Killing Assay and Effect of Ca^2+^ and Mg^2+^ on the Killing Activity of Pf-10*). A value of *p* ≤ 0.05 was considered statistically significant.

## 3. Results and Discussion

### 3.1. Phage Isolation, Host Range and Morphology

**Figure 1:**
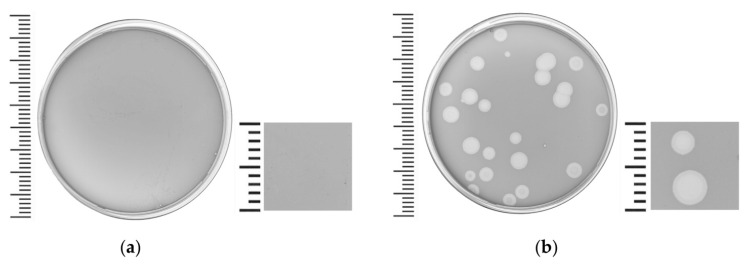
*Pseudomonas* phage Pf-10 plaque morphology on 0.75% (*w*/*v*) LB agar overlay. (**a**) Control plate: non-infected sensitive strain *P. syringae* BIM B-268. (**b**) Plaques formed by the Pf-10 phage on the lawn of strain *P. syringae* BIM B-268.

Phage Pf-10 was isolated in 2014 from a sample of green bean (*Phaseolus vulgaris*) tissue infected with *P. syringae* pv. *syringae* [40]. The Pf-10 phage was kindly provided by the Belarusian collection of non-pathogenic microorganisms (BIM) of the Institute of Microbiology, National Academy of Sciences, Minsk, Belarus. On the lawn of the sensitive strain *P. syringae* BIM B-268, the phage Pf-10 formed clear plaques approximately 4–8 mm in diameter with a characteristic small zone of incomplete lysis (Figure 1). Host range analysis revealed that Pf-10 was able to lyse 13 (28.9%) out of 45 strains (Supplementary Information, Table S1). Seven of the thirteen strains susceptible to the Pf-10 phage (Supplementary Information, Table S1) are considered non-pathogenic, including the *P. fluorescens* BIM B-582 strain used for the industrial production of the “Multiphage” phages [39]. The remaining six strains are phytopathogenic pseudomonads: *P. syringae* BIM B-268, *P. yamanorum* BIM B-1235, *P.* sp. BIM B-272, *P. syringae* BIM B-1144, *P. syringae* BIM B-1229 and *P. syringae* BIM B-1140 (Supplementary Information, Table S1). Moreover, the latter three pathogenic strains infect cucumber fruits (*Cucumis sativus* L.). It should be noted that the “Multiphage” containing the Pf-10 phage is registered as a biopesticide against *Pseudomonas*-associated cucumber infections [39].

**Figure 2:**
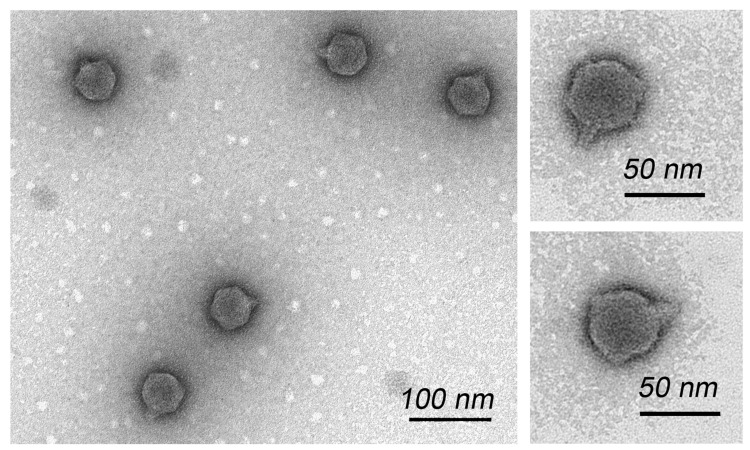
Transmission electron microscopy of *Pseudomonas* phage Pf-10 negatively stained with 1% (*w*/*v*) uranyl acetate. Scale bar: 100 nm and 50 nm.

Morphological analysis via TEM showed that phage Pf-10 had an icosahedral capsid of approximately 55.5 ± 2.8 nm in diameter and a very short, noncontractile tail of approximately 11.2 ± 0.8 nm in length (Figure 2). Electron microscopic analyses demonstrated that phage Pf-10 has the typical morphological features of the podovirus morphotype (Figure 2).

### 3.2. pH and Thermal Stability Tests

The loss of phage activity during storage, transfer, or phage inactivation due to in vivo environment (e.g., pH) may result in poor outcomes in case of using phage-based preparations in phage therapy or bacteriophage-based biocontrol. This issue requires attention in the area of phage formulation to ensure phage viability and stability during storage and use.

**Figure 3:**
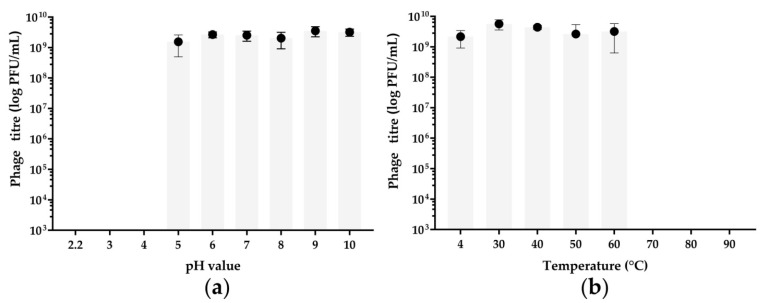
Thermal and pH stability of *Pseudomonas* phage Pf-10. (**a**) Phage survival after 1-h incubation at pH values ranging from 2.2 to 10. (**b**) Phage survival after 1-h incubation at the temperatures ranging from 4 to 90 °C. The graphs were created with GraphPad Prism 8.4.3. Values represent the mean of three independent trials, and error bars represent the standard deviation.

Phage Pf-10 was highly stable at pH values from 5 to 10 (Figure 3a) (comparison with a sample at pH 7 as a control; ANOVA, *p* > 0.05), while under highly acidic conditions (pH 2.2, 3, and 4), no phages survived after incubation (Figure 3a) (ANOVA, *p* < 0.05).

The thermal stability tests showed that Pf-10 was highly stable at temperatures of 30–60 °C (ANOVA, *p* > 0.05), as the phage titer was similar to those of the control sample incubated at 4 °C (Figure 3b). Phage Pf-10 completely lost its activity after 1-h incubation at 70 °C and higher temperatures (ANOVA, *p* < 0.05), as no plaques were detected after the incubation under these temperature conditions (Figure 3b).

### 3.3. Killing Assay and the Effect of Ca^2+^ and Mg^2+^ on the Killing Activity of Pf-10

One of the important phage characteristics that can be useful for choosing phages for phage-based preparations is the killing activity, also called the lytic activity of a phage. Analysis of the growth kinetics of a bacterial host upon phage infection is the simplest method for assessing the effectiveness of the killing activity of a phage. The rate of the OD curve slope is directly associated with the lytic activity and the concentration of the phage used. Various phages are characterized by a relatively high or low efficiency of their lytic activity, depending on the time and the magnitude of the decrease in OD of the bacterial culture upon infection.

**Figure 4:**
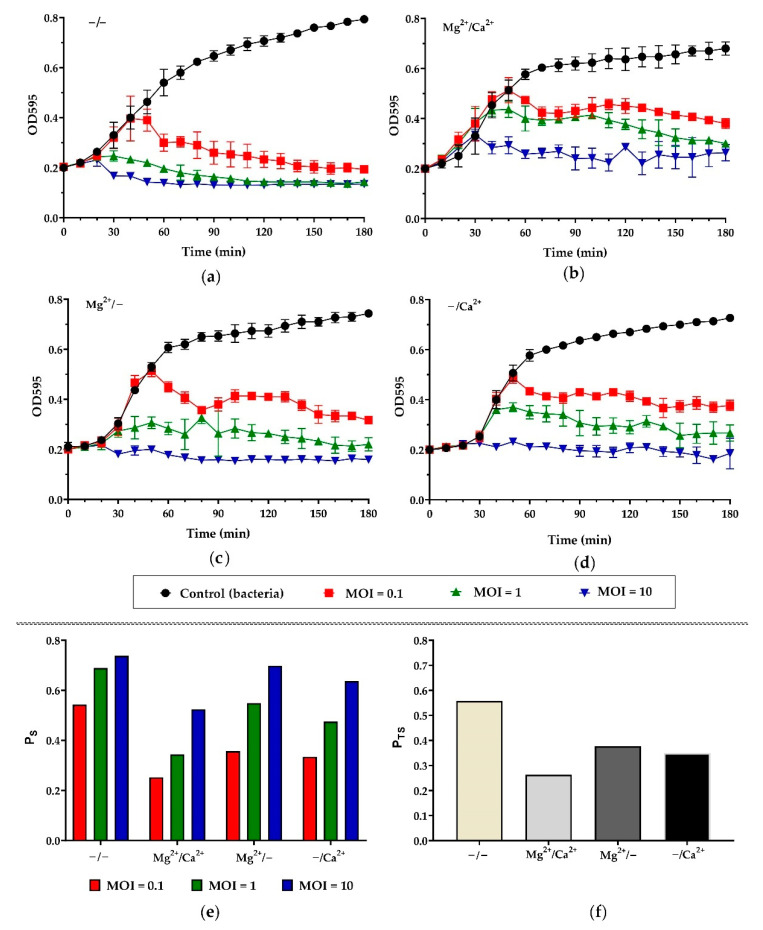
*P. syringae* BIM B-268 growth kinetics upon the infection with *Pseudomonas* phage Pf-10 at different MOI values. Killing activity of phage Pf-10 in: (**a**) the absence of Mg^2+^ and Ca^2+^ ions; (**b**) the presence of Mg^2+^ and Ca^2+^ ions; (**c**) the presence of only Mg^2+^ ions; (**d**) the presence of only Ca^2+^ ions. The graphs were created with GraphPad Prism 8.4.3. Values represent the mean of three independent trials, and error bars represent the standard deviation. (**e**) PhageScore factor (P_S_) and (**f**) Phage Total Score factor (P_TS_) values calculated for *Pseudomonas* phage Pf-10 for experiment the “*Killing Assay and Effect of Ca^2+^ and Mg^2+^ on the Killing Activity of Pf-10*”.

The killing activity of phage Pf-10 was assessed at MOI values of 0.1, 1 and 10 by measuring OD_595_ of infected *P. syringae* BIM B-268 cultures (Figure 4). The non-infected cultures were used as a control (Figure 4). Simultaneously, the effects of Ca^2+^ and Mg^2+^ on the killing activity of Pf-10 was assessed by adding CaCl_2_ and/or MgCl_2_ to the *P. syringae* BIM B-268 (Figure 4b–f) and comparing with the culture without the addition of the ions (Figure 4a,e,f).

Figure 4 shows that the inhibitory effect of phage Pf-10 on bacterial growth increases significantly in a MOI-dependent manner compared to the control (Figure 4a–d; for each graph ANOVA, *p* < 0.05). The statistical analyses showed significant differences between bacterial growth curves under Pf-10 infection in the presence and absence of Mg^2+^ and/or Ca^2+^ ions (for each MOI ANOVA, *p* < 0.05). As shown in Figure 4, the lysis of bacterial cells is more efficient without the addition of the ions at each MOI. The PhageScore method based on bacterial biomass concentration changes, which are directly related to the lytic potential of the tested phage, was used to assess the lytic activity of phage Pf-10 under various salt conditions. The PhageScore method is a simple method recently developed by Konopacki and co-authors for comparative evaluation of lytic activity of phages, which is measured in PhageScore factor values (P_S_) [44]. The proposed method makes it possible to accurately compare and analyze the activity of bacteriophages, therefore it can be applied for comparative analysis and selection of a proper phage for specific phage-based preparations. We believe that this method will find application for assessing the phage lytic activity under various medium conditions. Estimation of phage killing activity in an environment, similar to that where the phage-based preparation will be used, is an important step in the selection of candidate phages. The PhageScore values (P_S_) of Pf-10 upon infection at MOI of 0.1 and 1 are clearly reduced in the presence of MgCl_2_ and CaCl_2_ compared to the infection without additional Ca^2+^ and Mg^2+^ ions (Figure 4e). At MOI of 10, the Pf-10 P_S_ values decrease slightly under various conditions (Figure 4e). In addition, the Pf-10 Total PhageScore values have also been calculated (Figure 4f). As described by Konopacki [44], the Total PhageScore factor (P_ST_) is a parameter that characterizes the total lytic activity of a specific bacteriophage across the MOI range, being sensitive to the efficiency of the lowest dilution of the phage. Figure 4f shows that the Pf-10 P_ST_ decreases in the presence of: Mg^2+^ by 1.5 times, Ca^2+^ by 1.6 times and both Mg^2+^ and Ca^2+^ by 2.1 times. Thus, the presence of Ca^2+^ and Mg^2+^ ions can reduce the killing activity of the phage Pf-10.

### 3.4. Adsorption Assay and One-Step Growth Curve

The data on the adsorption constant, adsorption rate, latent period, and burst size are the most important parameters that can affect the success of phage-based preparations. Understanding the interaction of phages and bacteria (including the parameters of phage infection, as well as the conditions under which the infection occurs) can help us optimize phage production, develop effective protocols for the use of phage-based preparations, determine the optimal dose concentrations and prognosticate the results of phage therapy [60,61,62].

**Figure 5:**
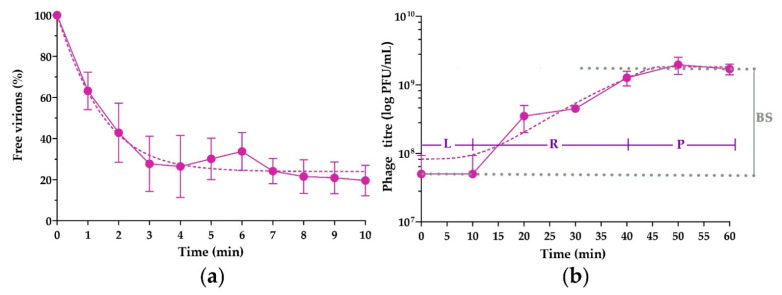
Infection parameters of *Pseudomonas* phage Pf-10. (**a**) Adsorption assay (**b**) One-step growth curve. L, latent phase; R, virion release phase; P, plateau phase; BS, burst size. The graphs were created with GraphPad Prism 8.4.3. Values in panels (**a**,**b**) represent the mean of five and three independent trials, respectively, and error bars represent the standard deviation.

Adsorption assay revealed that the adsorption time of Pf-10 required for the attachment of about 80% of the phage particles to host cells was 10 min (Figure 5a). The adsorption constant of phage Pf-10 calculated using the equation described by Kropinski [45] is (3.59 ± 1.42) × 10^−9^ mL/min. The adsorption rate of phage Pf-10 was similar to that of other reported *Pseudomonas*-infecting phages of the *Autographiviridae* family, namely: *Pseudomonas* phage Andromeda (1.48 × 10^−9^ mL/min) [63] and *Pseudomonas* phage PAXYB1 (3.70 × 10^−9^ mL/min) [64].

The one-step growth curve assay showed the main parameters of the phage infection: the Pf-10 latent period (virion maturation) is 10–20 min, the duration of the virion release phase is about 30 min. The average burst size of the phage per infected cell is 37 ± 8.5 PFU. The latency period of the Pf-10 phage was nearly similar to *Pseudomonas* phage gh-1 (21 min) [65], and shorter than that of most *Pseudomonas* phages of the *Autographiviridae* family, namely *Pseudomonas* phage Andromeda (25–30 min) [63], *Pseudomonas* phage PAXYB1 (30 min) [64] and *Pseudomonas* phage RLP (25 min) [66], and especially of *Pseudomonas* phage BF7, which shows an extremely long latent period (140 min) [67]. Interestingly, the burst size of Pf-10 was smaller than that of other reported *Pseudomonas* phages of the *Autographiviridae* family such as *Pseudomonas* phage gh-1 (103 PFU) [65], *Pseudomonas* phage Andromeda (70 PFU) [63], *Pseudomonas* phage PAXYB1 (141 PFU) [64], *Pseudomonas* phage RLP (154 PFU) [66] and *Pseudomonas* phage BF7 (237 PFU) [67]. However, it should also be noted that all time and kinetic parameters of phage adsorption and one-step growth curve may differ depending on the bacterial host. It is not impossible that a small number of Pf-10 progeny particles are associated with the intracellular metabolism of the *P. syringae* BIM B-268 strain.

### 3.5. General Genome Organization

**Figure 6:**
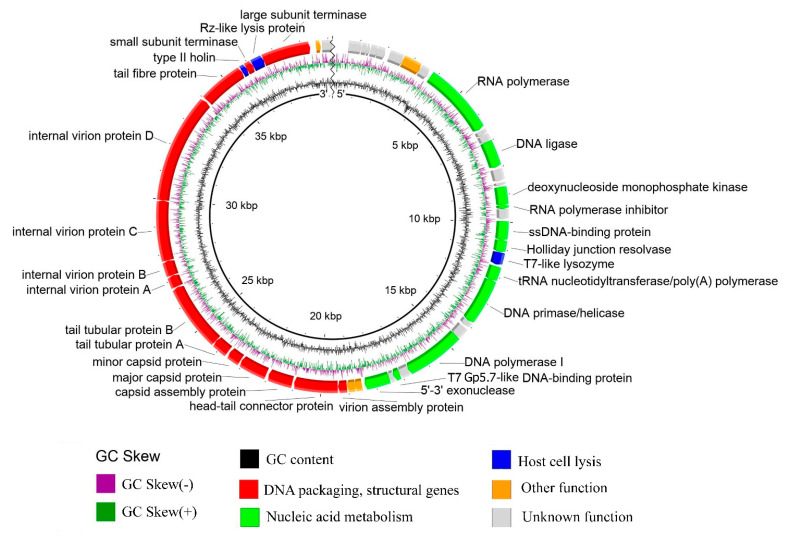
*Pseudomonas* phage Pf-10 genome map. Functionally assigned ORFs are highlighted according to their general functions (see the legend).

Genome sequencing and annotation have revealed that the Pf-10 genome is a linear dsDNA with the length of 39,167 bp (not including the second copy of the DTR) and the GC-content of 56.5%. The genome contains 49 genes (Supplementary Information, Table S2). Thirty two out of 49 predicted CDSs (65.3%) were functionally assigned using BLASTp (NCBI) [49] and HHpred [50] (Supplementary Information, Table S2). The schematic map of the phage genome is presented in Figure 6. The encoded proteins of phage Pf-10 are divided into several functional modules, the main of them being: DNA packaging and structure/morphogenesis module, lytic module, DNA replication and recombination module. It should be noted that the arrangement and structure of the Pf-10 modules resemble those in the *Enterobacteria* phage T7 (Supplementary Information, Figure S3).

#### 3.5.1. DNA Packaging Genes and the Genome Packaging Strategy

**Figure 7:**
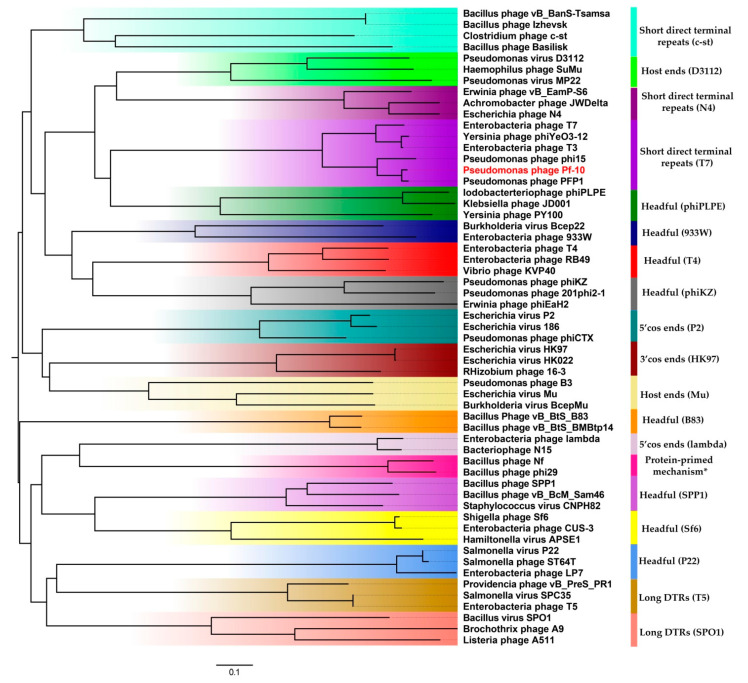
The phylogenetic tree based on the amino acid sequence of large terminase subunits using the proteins from the phage Pf-10 and from phages with well-known packaging mechanisms. The phylogenetic tree was constructed from MAFFT alignments using MEGA X by the neighbor-joining method with a bootstrapping of 100 and displayed using FigTree v1.4.4.

**Figure 8:**
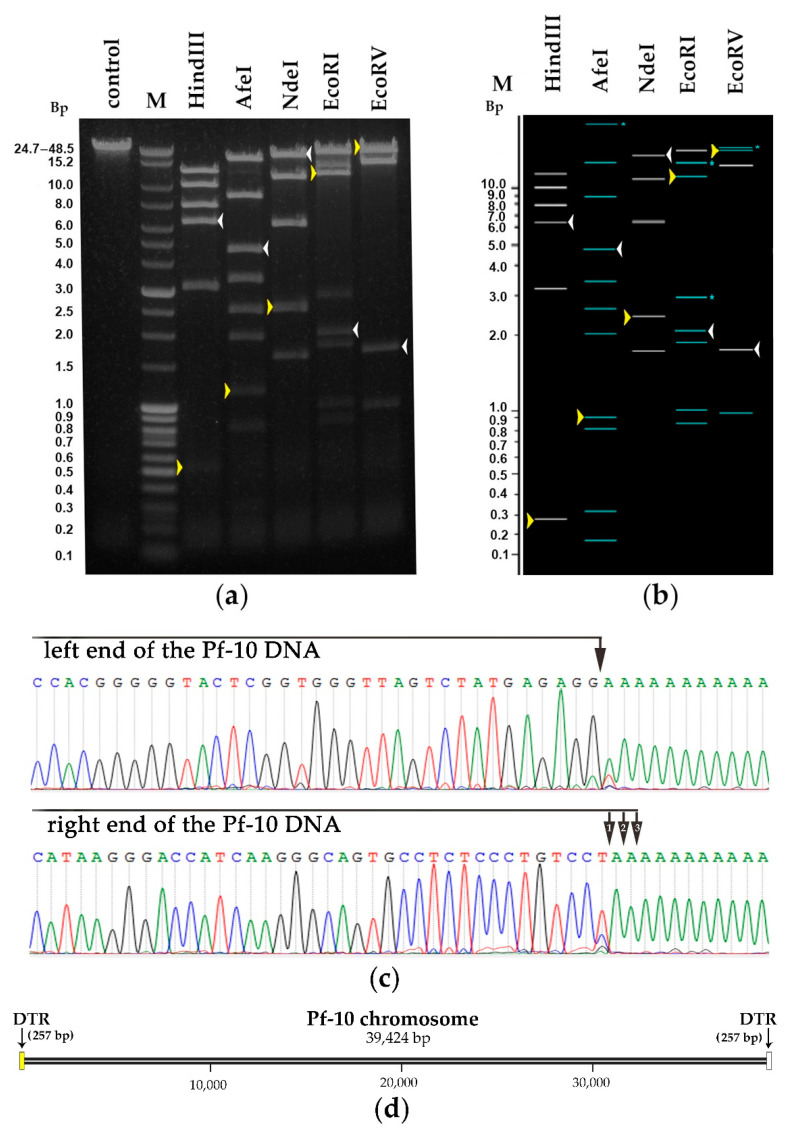
Determination of packaging mechanism and genome termini. Restriction analysis of the Pf-10 phage DNA: (**a**) in vitro and (**b**) in silico. In silico restriction digest of the original genome of phage Pf-10 (after genome assembly) was carried out using the NEBcutter V2.0 tool. M—molecular weight markers. The DNA-fragments containing the left and right ends of the phage DNA are indicated by yellow and white arrows, respectively in panels (**a**,**b**). The DNA-fragments with restriction pattern that can change if the cleavage is blocked by CpG methylation (in case of AfeI and EcoRI) and EcoBI methylation (in case of EcoRV) are light blue in panel (**b**). The original full-length image of the gel is presented in Supplementary Figure S4. (**c**) The terminal regions of the PCR product sequences obtained with RAGE for the right and left ends of the Pf-10 DNA are shown on the sequencing chromatograms. The tentative positions of the Pf-10 DNA ends, identified by RAGE method, are indicated with black arrows. (**d**) Schematic representation of the Pf-10 phage chromosome.

The DNA packaging module includes two proteins: a small terminase subunit (ORF45; protein_id YP_009145640.1) and a large terminase subunit (ORF47; protein_id YP_009145642.1). The critical step of the assembly of phages belonging to *Caudovirales* is known to be genome packaging performed by the large terminase subunit. In order to predict the type of genome termini and the packaging mechanism of phage Pf-10, the amino acid sequence of the large terminase protein was compared with the terminase protein from the published phages with well-studied DNA packaging mechanisms [68] (Supplementary Information, Table S4). As shown in Figure 7, the terminases of the phages: *Pseudomonas* phage Pf-10, *Pseudomonas* phage PFP1, *Pseudomonas* phage phi15, *Enterobacteria* phage T3, *Yersinia* phage phiYeO3-12 and *Enterobacteria* phage T7 form a separate clade, where the packaging protein of the phage Pf-10 is the closest to that of the phage PFP1 (Figure 7). Li, M. et al. (2018) have shown that the phage PFP1 DNA has short direct terminal repeats of 270 bp in length [69]. Restriction analysis was performed to identify the type of genome termini of phage Pf-10, as well as to confirm the completeness and correctness of the genome assembly. In silico restriction analysis of the Pf-10 genome was performed using the NEBcutter V2.0 tool [70] (Figure 8b). As shown in Figure 8, the restriction patterns for in vitro restriction analysis (Figure 8a) and in silico restriction analysis (Figure 8b) have some differences. It is clearly seen that in the case of restriction with HindIII, AfeI and NdeI, the DNA fragments containing the left end of the phage DNA (Figure 8, indicated with yellow arrow) are longer in vitro compared to their size in the in silico restriction analysis. In order to determine the ends of the Pf-10 genome more accurately, RAGE method was used, followed by Sanger sequencing. The analysis of the sequencing results revealed the presence of two short DTRs at the ends of the Pf-10 DNA (Figure 8c). Due to limitations of the RAGE method, it was difficult to determine the final nucleotide at the right end of the Pf-10 DNA using the method, since the sequence in the DTR-containing region had two consecutive adenine nucleotides (Figure 8c and Supplementary Information, Figure S5). Sanger sequencing of the right end of the Pf-10 DNA clarified the location of the right terminus (shown in Figure 8c by the second arrow). According to the results of phylogenetic and restriction analyzes, as well as sequencing of Pf-10 DNA ends, the phage uses a packaging mechanism based on T7-like short DTRs (Figure 7 and Figure 8), and the length of each terminal repeat in the phage chromosome is 257 bp (Figure 8d). Thus, the total length of the Pf-10 phage chromosome, including both DTRs, is 39,424 bp (Figure 8d).

#### 3.5.2. Structure/Morphogenesis and Lytic Genes

The module of structure/morphogenesis genes of phage Pf-10 as well as of other reported *Pseudomonas* phages of the genus *Pifdecavirus*, the *Autographiviridae* family resembles that of the lytic *Enterobacteria* phage T7 (Supplementary Information, Figure S3), which is well known to be the most studied member of the *Autographiviridae* family. The following 14 proteins of the phage Pf-10 were predicted to be involved in phage structure and morphogenesis (Figure 6; Supplementary Information, Table S2): virion assembly protein (ORF32; YP_009145627.1), head-tail connector protein (ORF33; YP_009145628.1), capsid assembly protein (ORF34; YP_009145629.1), major capsid protein (ORF35; YP_009145630.1), minor capsid protein (ORF36; YP_009145631.1), tail tubular protein A (ORF37; YP_009145632.1), tail tubular protein B (ORF38; YP_009145633.1), internal virion protein A (ORF39; YP_009145634.1), internal virion protein B (ORF40; YP_009145635.1), internal virion protein C (ORF41; YP_009145636.1), internal virion protein D (ORF42; YP_009145637.1), tail fiber protein (ORF43; YP_009145638.1), small terminase subunit (ORF45; YP_009145640.1), large terminase subunit (ORF47; YP_009145642.1) (Figure 6; Supplementary Information, Table S2 and Figure S3).The first step in a phage infection is recognition and binding to the bacterial host receptors, which is mediated by the receptor-binding proteins of the phage. The tail fiber proteins of *Pseudomonas* phages are receptor-binding proteins which facilitate phage entry into the host cell through recognition of bacterial outer membrane receptors. Different tail fiber proteins of *Pseudomonas* phages result in various host range. Therefore, comparing these proteins from different phages can contribute to the selection of phages for phage-based preparations. A BLASTp search showed that the tail fiber protein of the Pf-10 phage shared considerable similarity to tail fiber proteins of various phages, namely: *Pseudomonas* phage phiIBB-PF7A (protein_id YP_004306354.1; protein identity (PI) 85.64%; query coverage (QC) 100%) effective against *P. fluorescens* biofilms [71]; *Pseudomonas* phage Phi-S1 (YP_007869919.1; PI 66.07%; QC 56%) active against a broad range of fluorescent *Pseudomonas* species [72]; *Pseudomonas* phages 22PfluR64PP (YP_009801161.1), 67PfluR64PP (AWH15809.1) and 71PfluR64PP (AWH14721.1) (for each PI 68.81%; QC 39%) infecting *P. fluorescens* [73]. Phage lytic proteins are of similar interest as receptor-binding proteins. Lytic proteins are crucial for the host cell destruction during the burst stage of the phage life cycle. The lysis is mediated by the so-called two-component lysis systems, which comprise two proteins: a pore-forming holin and a cell wall degrading endolysin. These proteins can be obtained separately from phages and used as potential agents to inhibit or lyse pathogens. The lytic module of the Pf-10 phage consists of a T7-like lysozyme (ORF20; YP_009145615.1), a type II holin (ORF43; YP_009145639.1) and a Rz-like lysis protein (ORF46; YP_009145641.1) (Figure 6; Supplementary Information, Table S2 and Figure S3). The holin of Pf-10 is highly similar to the holins of the following phages: phage phiIBB-PF7A (YP_004306355.1; PI 100%; QC 100%); phage Phi-S1 (YP_007869920.1; PI 98.41%; QC 94%); phage 22PfluR64PP (YP_009801160.1; PI 93.65%; QC 94%). The T7-like lysozyme of Pf-10 is highly similar to: N-acetylmuramoyl-L-alanine amidase of phage Phi-S1(ID YP_007869897.1; PI 100%; QC 100%); putative lysozyme/amidase of phage phiIBB-PF7A (YP_004306332.1; PI 100%; QC 100%); putative lysozyme/amidase of phages 67PfluR64PP (AWH15781.1; PI 78.67%; QC 98%); 71PfluR64PP (AWH14698.1; PI 78%; QC 98%) and 22PfluR64PP (YP_009801184.1; PI 77.33%; QC 98%). The Rz-like lysis protein of the Pf-10 phage is highly identical to proteins such as putative cell lysis protein/endopeptidase of phage phiIBB-PF7A (YP_004306357.1; PI 97.93%; QC 100%); Rz-like lysis protein of phage Phi-S1 (YP_007869922.1; PI 88.28%; QC 100%); endopeptidases of phages 22PfluR64PP (YP_009801158.1), 67PfluR64PP (AWH15806.1) and 71PfluR64PP (AWH14724.1) (for each PI 75.17%; QC 100%). Unfortunately, the lytic proteins of the listed phages have not yet been studied.

#### 3.5.3. Replication Genes

The module of replication-related genes of phage Pf-10 resembles that of the lytic *Enterobacteria* phage T7 (Supplementary Information, Figure S3) and belongs to the “T7-type replication module” according to the classification proposed by Weigel and Seitz [74]. The replication module of phage Pf-10 consists of T7-like DNA-directed RNA polymerase (ORF9; YP_009145605.1), ATP-dependent DNA ligase (ORF12; YP_009145608.1), single-stranded DNA-binding protein (ORF18; YP_009145613.1), Holliday junction resolvase (ORF19; YP_009145614.1), T7 Gp4D-like DNA primase/helicase (ORF22; YP_009145617.1), DNA polymerase I (ORF25; YP_009145620.1) and 5′-3′ exonuclease (ORF29; YP_009145624.1) (Figure 6; Supplementary Information, Table S2 and Figure S3).

### 3.6. Comparative Genomics

**Figure 9:**
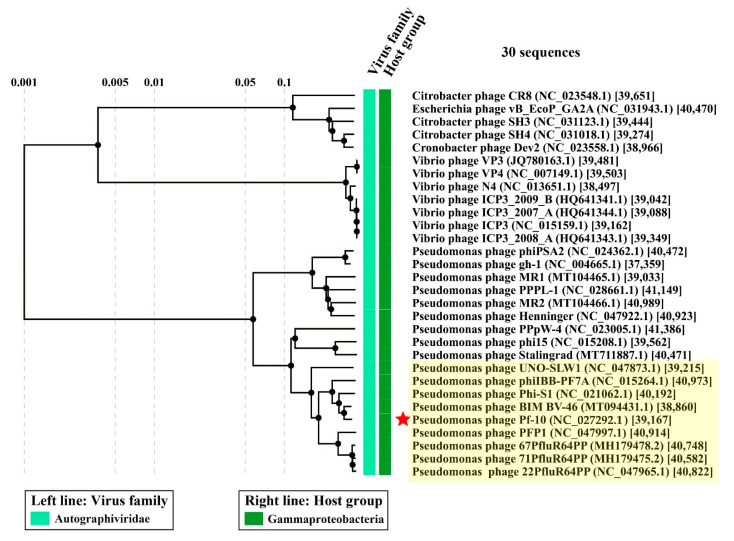
The viral proteomic tree including *Pseudomonas* phage Pf-10 and other most closely related phages constructed using the ViPTree server version 1.9. The red star represents the *Pseudomonas* phage Pf-10. The clade containing the genus *Pifdecavirus* phages is highlighted in light yellow.

**Figure 10:**
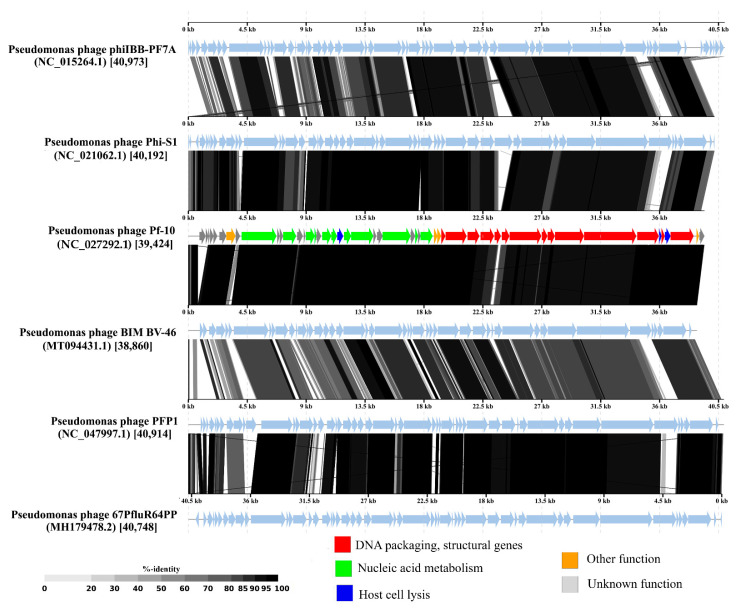
The pairwise tBLASTx comparison of the complete genomic DNA sequences of *Pseudomonas* phage Pf-10 and the most closely related phages visualized with ViPTree server version 1.9. The Pf-10 genome color scheme corresponds to Figure 6 (see the legend). Gray areas between the genome maps indicate the level of identity.

In order to assess the phylogenetic relationship between the phage Pf-10 and the known phages, the viral proteomic tree was constructed using the ViPTree server version 1.9. The viral proteomic tree showed that the closest relative of the phage Pf-10 was the *Pseudomonas* phage BIM BV-46, which, as with Pf-10, is part of the biopesticide “Multiphage” (Figure 9). According to the BLASTn (NCBI), the whole genome identity of Pf-10 and BIM BV-46 is 93.5% (Supplementary Information, Table S5), indicating that they are the different phage species in accordance with the official ICTV classification (the species demarcation criterion is the genome nucleotide identity of 95%) [75]. As shown in Figure 9, phage Pf-10 forms a common giant branch with the *Pseudomonas* phages. However, Pf-10 has more than 70% of the whole genome identity with only eight phages from this huge branch, namely, phage BIM BV-46, phage Phi-S1, phage phiIBB-PF7A, phage PFP1, phage 22PfluR64PP, phage 67PfluR64PP, phage 71PfluR64PP, and phage UNO-SLW1 (Figure 9; Supplementary Information, Table S5), which indicates that these phages belong to the same phage genus in accordance with the genus demarcation criterion of the official ICTV classification [75]. The phage DNA comparison diagram visualized using the ViPTree server version 1.9 shows the tBLASTx pairwise similarities between the most closely related phage genomic DNA (Figure 10).

Thus, phage Pf-10 can be classified as a new phage species belonging to the genus *Pifdecavirus*, the subfamily *Studiervirinae*, the family *Autographiviridae*, and the order *Caudovirales*.

## 4. Conclusions

In summary, we have described a new bacteriophage Pf-10 isolated from a tissue sample of green bean (*Phaseolus vulgaris*) infected with the *P. syringae* pv. *syringae*. The *Pseudomonas* phage Pf-10, along with five other bacteriophages of the *Pseudomonas* genus, is a part of the biopesticide “Multiphage” [39], which is used in Belarus to control the bacterial diseases of agricultural crops caused by *Pseudomonas syringae*. The phage Pf-10 is the first phage from the “Multiphage” that has been sequenced and subjected to a thorough analysis, including TEM analysis, stability test, genome analyses (the genome organization and the genome packaging strategy), phylogenetic analysis and determination of phage infection parameters such as the rate of adsorption, the life cycle and size of phage progeny on the *P. syringae* BIM B-268. The results of the study contribute to the accumulation of data on phage biology that can help in formulation of the most efficient phage combinations in phage-based preparations to combat plant diseases caused by *Pseudomonas*.

## Data Availability

The Pf-10 phage genome sequence was deposited into the GenBank database under the accession number KP025626 and NCBI Reference Sequence database under the accession number NC_027292.1 (BioProject accession number PRJNA263998, BioSample accession number SAMN21529907). The raw sequence dataset is available at the Sequence Read Archive under the accession number SRR15990561.

## Supplementary Materials

The following are available online at https://www.mdpi.com/article/10.3390/v14010042/s1. Table S1: *Pseudomonas* strains used in this study and infectivity of phage Pf-10; Figure S1: Transmission electron microscopy of the *Pseudomonas* phage Pf-10; Figure S2: Transmission electron microscopy of the *Pseudomonas* phage Pf-10; Table S2: Annotation of *Pseudomonas* phage Pf-10; Table S3: Primers used to locate the ends of the Pf-10 genome; Table S4: Large terminase subunit of Pf-10 and large terminase subunits of phages with well-known packaging mechanisms used for the phylogenetic tree; Figure S3: The pairwise whole-genome tBLASTx comparison of *Pseudomonas* phage Pf-10 and *Enterobacteria* phage T7 visualized with ViPTree server version 1.9; Table S5: Phage genomes used for phylogenetic inference; Figure S4: Restriction analysis of the phage genomic DNA; Figure S5: Schematic representation of the DTR-containing region of the Pf-10 genome after whole genome sequencing and genome assembly.
