# Supplementary material for: The Bacteriophage Pf-10—A Component of the Biopesticide “Multiphage” Used to Control Agricultural Crop Diseases Caused by Pseudomonas syringae
Source: Viruses. 2021 Dec 27;14(1):42. doi: 10.3390/v14010042 (PMC8779105; doi:10.3390/v14010042)
Supplement: Supplementary file 1 [file viruses-14-00042-s001.zip › viruses-1424299-supplementary.pdf]

The bacteriophage Pf-10 – a component of the biopesticide "Multiphage" used to control agricultural crop diseases caused by *Pseudomonas syringae*

Olesya A. Kazantseva [1,*], Rustam M. Buzikov [1], Tatsiana A. Pilipchuk [2], Leonid N. Valentovich [2,3], Andrey N. Kazantsev [4], Emilia I. Kalamiyets [2], and Andrey M. Shadrin [1]

[1] Laboratory of Bacteriophage Biology, G. K. Skryabin Institute of Biochemistry and Physiology of Microorganisms, Pushchino Scientific Center for Biological Research of the Russian Academy of Sciences, Federal Research Center, Pushchino, Moscow Oblast, Russia
[2] Institute of Microbiology of the National Academy of Sciences of Belarus, Minsk, Belarus
[3] Faculty of Biology, Belarusian State University, Minsk, Belarus
[4] P. N. Lebedev Physical Institute of the Russian Academy of Sciences, Pushchino Radio Astronomy Observatory, Pushchino, 142290, Russia

Supplementary Information:

[*] Correspondence: olesyakazantseva@bk.ru (O.A.K.); andrey2010s@gmail.com (A.M.S.)

Table S1. *Pseudomonas* strains used in this study and infectivity of phage Pf-10.

№	Bacterial species	Strain	Phage lysis
1	*P. amygdali* pv. *lachrymans*	BIM B-695	-
2	*P. cerasi*	BIM B-280	-
3	*P. corrugata*	BIM B-627	-
4	*P. corrugata*	BIM B-628	-
5	*P. corrugata*	BIM B-673	-
6	*P. congelans*	BIM B-240	-
7	*P. cremoricolorate*	BIM B-247	-
8	*P. ficuserectae*	BIM B-239	-
9	*P. frederiksbergensis*	BIM B-159	+
10	*P. fluorescens*	BIM B-147	+
11	*P. fluorescens*	BIM B-158	-
12	*P. fluorescens*	BIM B-186	-
13	*P. fluorescens*	BIM B-188	-
14	*P. fluorescens*	BIM B-582	+
15	*P. fluorescens*	BIM B-1132	-
16	*P. fluorescens*	BIM B-1143	+
17	*P. helmanticensis*	BIM B-86	+
18	*P. koreensis*	BIM B-1139	+
19	*P. putida*	BIM B-225	-
20	*P. putida*	BIM B-227	-
21	*P. putida*	BIM B-749	-
22	*P. putida*	BIM B-1142	+
23	*P. syringae*	BIM B-266	-
24	*P. syringae*	BIM B-267	-
25	*P. syringae*	BIM B-268	+
26	*P. syringae*	BIM B-833	-
27	*P. syringae*	BIM B-852	-
28	*P. syringae*	BIM B-853	-
29	*P. syringae*	BIM B-855	-
30	*P. syringae*	BIM B-1136	-
31	*P. syringae*	BIM B-1137	-
32	*P. syringae*	BIM B-1140	+
33	*P. syringae*	BIM B-1144	+
34	*P. syringae*	BIM B-1229	+
35	*P. yamanorum*	BIM B-1235	+
36	*P.* sp	BIM B-68	-
37	*P.* sp	BIM B-99	-
38	*P.* sp	BIM B-151	-
39	*P.* sp	BIM B-152	-
40	*P.* sp	BIM B-164	-
41	*P.* sp	BIM B-272	+
42	*P.* sp	BIM B-273	-
43	*P.* sp	BIM B-274	-
44	*P.* sp	BIM B-634	-
45	*P.* sp	BIM B-748	-

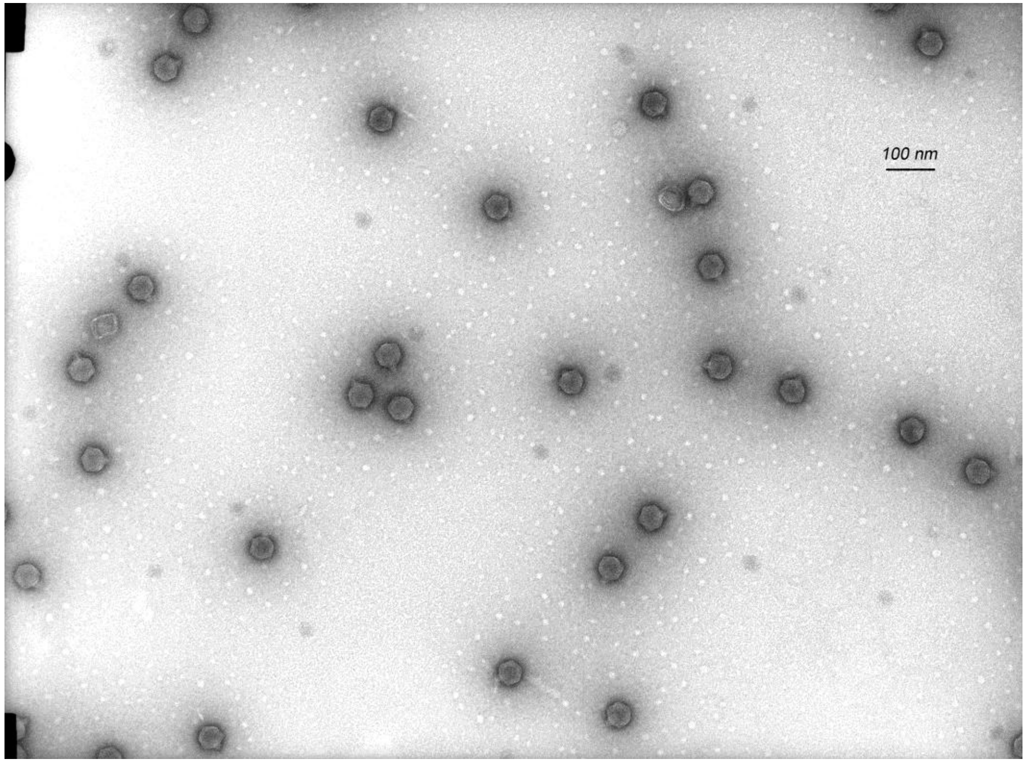

Figure S1. Transmission electron microscopy of the *Pseudomonas* phage Pf-10. This is the original TEM micrograph used to generate Figure 1 in the main text. The image was taken using a JEM 1200EX (JEOL, Japan) transmission electron microscope at 80 kV accelerating voltage on Kodak film SO-163 (Kodak, Cat. # 74144, Hatfield, PA, USA).

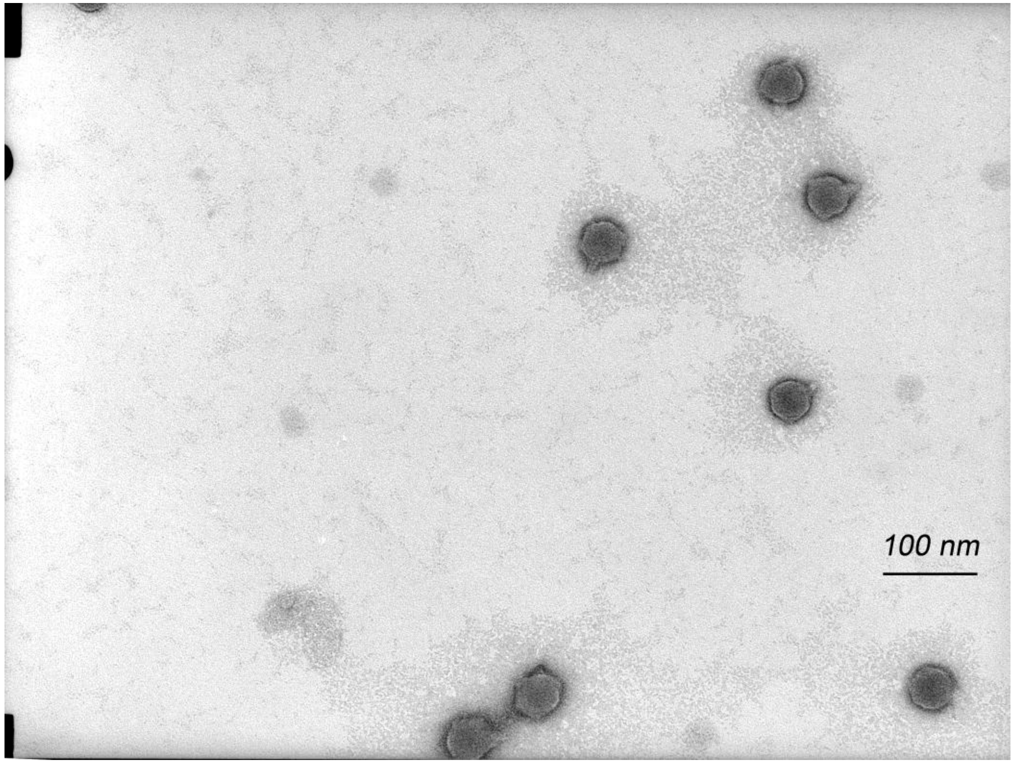

Figure S2. Transmission electron microscopy of the *Pseudomonas* phage Pf-10. This is the original TEM micrograph used to generate Figure 1 in the main text. The image was taken using a JEM 1200EX (JEOL, Japan) transmission electron microscope at 80 kV accelerating voltage on Kodak film SO-163 (Kodak, Cat. # 74144, Hatfield, PA, USA).

Table S2. Annotation of *Pseudomonas* phage Pf-10

ORF№	Start codon	Stop codon	Strand	Blast results		Conserved domains, Blast		Hhpred results	Annotation
				Name	E-val	Name, (region)	E-val	(Prob./E-val)	
1	588	1070	+	hp				COG5031; COQ4; Ubiquinone biosynthesis protein Coq4 [Coenzyme transport and metabolism].(94.08/1.30e-01)	hp
2	1070	1273	+	hp				('-', '-')	hp
3	1263	1385	+	hp				d1neea2; g.59.1.1 (A:99-135) Zinc-binding domain of translation initiation factor 2 beta {*Methanobacterium thermoautotrophicum* [T(61.33/6.20e-01)	hp
4	1398	1673	+	virion structural protein [*Pseudomonas* phage phi15]	9.0e-08	-		('-', '-')	hp
5	1657	1956	+	hp				PF13253.8; DUF4044 ; Protein of unknown function (DUF4044)(92.31/6.00e-01)	hp
6	2112	2648	+	virion structural protein [*Pseudomonas* phage phi15]<br><br>membrane protein [*Pseudomonas* phage Waldo5]<br><br>membrane protein	3.0e-40<br><br>9.0e-11<br><br>3.0e-10	-		('-', '-')	hp

				[*Pseudomonas* phage PlaquesPlease]					
7	2645	3349	+	putative 20G Fe(II) oxygenase [*Pseudomonas* phage BIM BV-46]<br><br>structural protein [*Pseudomonas* phage Stalingrad]<br><br>glycosyltransferase [*Pseudomonas* phage Waldo5]	4.0e-170<br><br>2.0e-88<br><br>7.0e-69	P4Hc (138-230)	1.39e-10	KOG1591; Prolyl 4-hydroxylase alpha subunit [Amino acid transport and metabolism](99.82/2.30e-19)<br><br>d2jiga_; b.82.2.15 (A:) P4H-1 {Green alga (*Chlamydomonas reinhardtii*) [TaxId: 3055]}(99.82/4.70e-19)<br><br>PF03336.15; Pox_C4_C10 ; Poxvirus C4/C10 protein(99.56/5.20e-14)	Prolyl 4-hydroxylase alpha subunit homologue
8	3353	3694	+	glycosyltransferase [*Pseudomonas* phage Stalingrad]	6.0e-07	-		('-', '-')	hp
9	3814	6468	+	DNA-directed RNA polymerase [*Pseudomonas* phage Phi-S1]<br><br>RNA polymerase [*Pseudomonas* phage phiIBB-PF7A]	0.0<br><br>0.0			d1mswd_; e.8.1.3 (D:) T7 RNA polymerase {Bacteriophage T7 [TaxId: 10760]}(100.0/1.30e-164)<br><br>KOG1038; Mitochondrial/chloroplast DNA-directed RNA polymerase RPO41, provides primers for DNA replication-initiation [Transcript(100.0/1.20e-148)<br><br>COG5108; RPO41; Mitochondrial DNA-directed RNA polymerase [Transcription].(100.0/2.80e-147)	T7-like DNA-directed RNA polymerase
10	6531	6722	+	hp				PF08200.13; Phage_1_1 ;	hp

								Bacteriophage 1.1 Protein(96.81/7.50e-04)	
11	6719	6991	+	hp				('-', '-')	hp
12	6991	7974	+	DNA ligase [*Pseudomonas* phage BIM BV-46]<br><br>putative DNA ligase [*Pseudomonas* phage phiIBB-PF7A]	0.0e+00<br><br>0.0e+00	PHA00454 (5-320)<br><br>DNA_ligase_C (230-318)<br><br>CDC9 (2-318)	4.09e-107<br><br>8.15e-15<br><br>2.83e-08	KOG0967; ATP-dependent DNA ligase I [Replication, recombination and repair](100.0/1.70e-36)<br><br>COG1793; CDC9; ATP-dependent DNA ligase [Replication, recombination and repair].(100.0/4.00e-36)<br><br>KOG0966; ATP-dependent DNA ligase IV [Replication, recombination and repair](100.0/8.40e-33)	ATP-dependent DNA ligase
13	8051	8497	+	DNA ligase [*Pseudomonas* phage Henninger]	1.0e-03	-		PF10669.11; Phage_Gp23 ; Protein gp23 (Bacteriophage A118)(90.55/1.50e+00)<br><br>PF06667.14; PspB; Phage shock protein B (90.28/3.9)	hp
14	8556	8684	+	-				COG3197; FixS; Cytochrome oxidase maturation protein, CcoS/FixS family [Posttranslational modification, protein turnover, chapero(89.75/1.80e+00)<br><br>cd19065 LGIC_TM_ZAC; transmembrane domain of zinc-activated ligand-gated ion channel. This family contains	hp

								transmembrane (TM) do (89.44/1.9)	
15	8714	9394	+	virion structural protein [*Pseudomonas* phage phi15]<br><br>deoxynucleoside monophosphate kinase [*Pseudomonas* phage PlaquesPlease]<br><br>structural protein [*Pseudomonas* phage Stalingrad]	3.0e-69<br><br>6.0e-58<br><br>4.0e-57	PHA02575 (97-165)	2.98e-03	d1deka_; c.37.1.1 (A:) Deoxynucleoside monophosphate kinase {Bacteriophage T4 [TaxId: 10665]}(99.87/2.50e-20)<br><br>PF04275.16; P-mevalo_kinase ; Phosphomevalonate kinase(98.78/1.20e-07)<br><br>d1vhta1; c.37.1.1 (A:2-206) Dephospho-CoA kinase {*Escherichia coli* [TaxId: 562]}(98.68/5.60e-07)	deoxynucleoside monophosphate kinase
16	9391	9528	+	RNA polymerase inhibitor [*Pseudomonas* phage PFP1]<br><br>bacterial RNA polymerase inhibitor [*Pseudomonas* phage phiIBB-PF7A]<br><br>putative bacterial RNA polymerase inhibitor [*Pseudomonas* phage PPpW-4]	2.0e-23<br><br>1.0e-21<br><br>2.0e-11	RNA_pol_inhib (2-42)<br><br>PHA00457 (13-43)	8.51e-10<br><br>7.03e-06	PF16857.7; RNA_pol_inhib ; RNA polymerase inhibitor(99.9/1.30e-23)<br><br>PF14207.8; DpnD-PcfM ; DpnD/PcfM-like protein(97.17/2.10e-03)	RNA polymerase inhibitor
17	9525	9896	+	hp				('-', '-')	hp
18	9970	10662	+	single-stranded DNA-binding protein [*Pseudomonas* phage BIM BV-46]	4.0e-169	PHA00458 (1-230)	4.85e-93	d1je5a_; b.40.4.7 (A:) gp2.5 {Bacteriophage T7 [TaxId: 10760]}(99.97/1.50e-29)	single-stranded DNA-binding protein

				single-stranded DNA-binding protein [*Pseudomonas* phage Phi-S1]	6.0e-169			PF10991.10; DUF2815 ; Protein of unknown function (DUF2815)(99.95/1.70e-26)	
19	10665	11102	+	endonuclease [*Pseudomonas* phage Phi-S1]<br><br>endonuclease I [*Pseudomonas* phage BIM BV-46]<br><br>endonuclease I [*Pseudomonas* phage PFP1]	6.0e-104<br><br>2.0e-103<br><br>2.0e-100	PHA00159 (1-145)<br><br>Phage_endo_I (1-145)	1.17e-75<br><br>1.95e-72	PF05367.13; Phage_endo_I ; Phage endonuclease I(100.0/9.00e-33)<br><br>d1m0da_; c.52.1.17 (A:) Endonuclease I (Holliday junction resolvase) {Bacteriophage T7 [TaxId: 10760]}(99.97/2.00e-29)<br><br>PF06356.13; DUF1064 ; Protein of unknown function (DUF1064)(99.2/2.50e-10)	Phage endonuclease I (Holliday junction resolvase)
20	11114	11575	+	N-acetylmuramoyl-L-alanine amidase [*Pseudomonas* phage Phi-S1]<br><br>lysin [*Pseudomonas* phage UNO-SLW1]<br><br>putative lysozyme/amidase [*Pseudomonas* phage phiIBB-PF7A]	1.0e-111<br><br>4.0e-94<br><br>6.0e-92	PHA00447 (4-149)<br><br>AmpD (49-136)	1.33e-72<br><br>8.79e-09	d1lbaa_; d.118.1.1 (A:) Bacteriophage T7 lysozyme (Zn amidase) {Bacteriophage T7 [TaxId: 10760]}(99.86/2.20e-19)<br><br>d2cb3a1; d.118.1.1 (A:174-344) Peptidoglycan-recognition protein-LE {Fruit fly (*Drosophila melanogaster*) [TaxId: 7227]}(99.82/3.40e-18)<br><br>d2f2lx1; d.118.1.1 (X:335-499) Peptidoglycan-recognition protein-LC {Fruit fly (*Drosophila melanogaster*) [TaxId: 7227]}(99.8/7.70e-18)	T7-like lysozyme

21	11641	12177	+	putative nucleotidyl transferase [*Pseudomonas* phage vB_PsyP_3MF5]<br><br>putative nucleotidyl transferase [*Pseudomonas* phage PPpW-4]	5.0e-50<br><br>4.0e-42	PHA01806 (22-158)	5.58e-18	KOG2159; tRNA nucleotidyltransferase/poly(A) polymerase [Translation, ribosomal structure and biogenesis](99.93/3.90e-25)<br><br>COG0617; PcnB; tRNA nucleotidyltransferase/poly(A) polymerase [Translation, ribosomal structure and biogenesis].(99.91/1.00e-23)<br><br>d1vfga2; d.218.1.4 (A:1-136) Poly A polymerase PcnB {*Aquifex aeolicus* [TaxId: 63363]}(99.87/1.30e-21)	tRNA nucleotidyltransferase/poly(A) polymerase
22	12181	13872	+	primase/helicase protein [*Pseudomonas* phage Phi-S1]<br><br>DNA primase/helicase protein [*Pseudomonas* phage PFP1]	0.0e+00<br><br>0.0e+00	RecA-like_Gp4D_helicase (302-528)<br><br>DnaB (258-532)<br><br>TOPRIM_primases (149-232)<br><br>ATPase (296-441)	1.16e-91<br><br>5.17e-11<br><br>1.34e-09<br><br>2.65e-06	KOG2373; Predicted mitochondrial DNA helicase twinkle [Replication, recombination and repair](100.0/2.90e-38)<br><br>d1cr1a_; c.37.1.11 (A:) Gene 4 protein (g4p, DNA primase), helicase domain {Bacteriophage T7 [TaxId: 10760]}(99.95/2.70e-24)<br><br>cd01122; GP4d_helicase; GP4d_helicase is a homohexameric 5'-3' helicases. Helicases couple NTP hydrolysis to the unwinding of nuc(99.86/7.00e-21)	T7 Gp4D-like DNA primase/helicase

23	13875	14093	+	virion structural protein [*Pseudomonas* phage phi15]	4.0e-09	-		PF04521.15; Viral_P18 ; ssRNA positive strand viral 18kD cysteine rich protein(85.39/3.60e+00)	hp
24	14152	14592	+	hp				d1cuka1; a.5.1.1 (A:156-203) DNA helicase RuvA subunit, C-terminal domain {*Escherichia coli* [TaxId: 562]}(75.22/3.20e+00)	hp
25	14579	16717	+	DNA polymerase [*Pseudomonas* phage Phi-S1]<br><br>DNA polymerase [*Pseudomonas* phage phiIBB-PF7A]	0.0e+00<br><br>0.0e+00	DNA_pol_A_pol_I_B (223-712)<br><br>DNA_pol_A (330-705)<br><br>PolA (39-712)	2.35e-149<br><br>2.46e-28<br><br>2.38e-10	COG0749; PolA; DNA polymerase I - 3'-5' exonuclease and polymerase domains [Replication, recombination and repair].(100.0/2.90e-69)<br><br>d1kfsa2; e.8.1.1 (A:519-928) DNA polymerase I (Klenow fragment) {*Escherichia coli* [TaxId: 562]}(100.0/1.80e-59)<br><br>d3py8a2; e.8.1.1 (A:423-832) DNA polymerase I (Klenow fragment) {*Thermus aquaticus* [TaxId: 271]}(100.0/2.00e-58)	DNA polymerase I (Klenow fragment)
26	16717	17076	+	hp				PF11247.10; ; DUF2675 ; Protein of unknown function (DUF2675)(99.95/1.90e-27)	hp
27	17079	17288	+	putative HNS binding protein [*Pseudomonas* phage MR2]<br><br>HNS binding protein [*Pseudomonas* phage UNO-SLW1]	4.0e-28<br><br>2.0e-27	PHA00422 (1-69)	2.16e-32	d2hgca1; a.4.5.77 (A:5-82) Uncharacterized protein YjcQ {Bacillus subtilis [TaxId: 1423]}(96.25/2.50e-02)<br><br>PF10711.11; DUF2513 ; Hypothetical protein (DUF2513)(96.26/3.00e-02)	T7 Gp5.7-like DNA-binding protein

				putative HNS binding protein [*Pseudomonas* phage MR1]<br><br>gene 5.7 [*Escherichia* phage T7]	2.0e-27<br><br>2e-20			PF14338.8; Mrr_N ; Mrr N-terminal domain (95.51/4.80e-02)<br><br>d2obpa1 a.4.5.71 (A:12-92) Putative DNA-binding protein ReutB4095 (96.23/0.11)	
28	17285	17488	+	hp				cd00731; CheA_reg; CheA regulatory domain; CheA is a histidine protein kinase present in bacteria and archea.(80.34/6.70e+00)	hp
29	17485	18399	+	exonuclease [*Pseudomonas* phage Phi-S1]<br><br>putative exonuclease [*Pseudomonas* phage phiIBB-PF7A]	0.0e+00<br><br>0.0e+00	PHA00439 (1-302)<br><br>53EXOc (86-210)	6.79e-127<br><br>9.03e-08	COG0258; Exo; 5'-3' exonuclease [Replication, recombination and repair].(99.93/4.70e-25)<br><br>d3h7ia1; c.120.1.2 (A:4-180) T4 RNase H {Bacteriophage T4 [TaxId: 10665]}(99.92/1.30e-24)<br><br>cd09859; PIN_53EXO; FEN-like PIN domains of PIN domain of the 5'-3' exonuclease of *Thermus aquaticus* DNA polymerase I (Taq) and h(99.87/1.40e-21)	5'-3' exonuclease
30	18508	18774	+	DUF2717 domain-containing protein [*Pseudomonas* phage 17A]<br><br>gp6.5 [*Escherichia* phage 285P]<br><br>gp6.5 [*Erwinia* phage vB_EamP-L1]	2.0e-26<br><br>6.0e-14<br><br>1.0e-12	PHA00438 (1-82)	6.57e-26	PF10911.10; DUF2717 ; Protein of unknown function (DUF2717)(100.0/8.70e-42)	DUF2717 domain-containing protein

31	18761	19051	+	virion structural protein [*Pseudomonas* phage 67PfluR64PP]<br><br>virion structural protein [*Pseudomonas* phage phi15]<br><br>putative virion protein [*Pseudomonas* phage 17A]	8.0e-26<br><br>1.0e-19<br><br>2.0e-17	DUF5476 (1-62)	1.44e-08	PF17570.4; DUF5476 ; Family of unknown function (DUF5476)(99.87/3.10e-22)	DUF5476 domain-containing protein
32	19061	19381	+	tail assembly protein [*Pseudomonas* phage phiIBB-PF7A]<br><br>tail assembly protein [*Pseudomonas* phage PFP1]	2.0e-32<br><br>1.0e-17	PHA00437 (22-106)<br><br>VirionAssem_T7 (65-106)	3.38e-10<br><br>2.34e-07	PF11653.10; VirionAssem_T7 ; Bacteriophage T7 virion assembly protein(99.98/3.60e-33)	virion assembly protein
33	19394	21001	+	head-tail connector protein [*Pseudomonas* phage Phi-S1]<br><br>head-tail connector protein [*Pseudomonas* phage phiIBB-PF7A]	0.0e+00<br><br>0.0e+00	Head-tail_con (18-477)	2.09e-84	PF12236.10; Head-tail_con ; Bacteriophage head to tail connecting protein(100.0/1.00e-45)<br><br>PF16510.7; P22_portal ; Phage P22-like portal protein(99.96/1.10e-26)<br><br>PF05133.16; Phage_prot_Gp6 ; Phage portal protein, SPP1 Gp6-like(99.68/3.60e-13)	head-tail connector protein (portal)
34	21067	21978	+	capsid assembly scaffolding protein [*Pseudomonas* phage BIM BV-46]	0.0e+00	PHA00435 (1-303)<br><br>Phage_T7_Capsid (149-250)	2.11e-106<br><br>4.14e-42	PF05396.13; Phage_T7_Capsid ; Phage T7 capsid assembly protein(99.94/2.70e-25)	capsid assembly protein

				capsid assembly protein [*Pseudomonas* phage Phi-S1]	0.0e+00				
35	22067	23092	+	minor capsid protein 10B [*Pseudomonas* phage phiIBB-PF7A]<br><br>major capsid protein 10A [*Pseudomonas* phage phiIBB-PF7A]	0.0e+00<br><br>0.0e+00	PHA00201 (1-341)	0e+00	PF19821.1; Phage_capsid_2 ; Phage capsid protein(99.92/6.00e-24)<br><br>PF11651.10; P22_CoatProtein ; P22 coat protein - gene protein 5(99.81/4.50e-18)<br><br>PF05065.15; Phage_capsid ; Phage capsid family(99.78/4.90e-18)	major capsid protein
36	23134	23622	+	minor capsid protein 10B [*Pseudomonas* phage phiIBB-PF7A]<br><br>minor capsid protein [*Pseudomonas* phage BIM BV-46]	3.0e-104<br><br>5.0e-31	YjdB (26-151)<br><br>Big_2 (86-153)	8.16e-10<br><br>9.34e-05	COG5492; YjdB; Uncharacterized conserved protein YjdB, contains Ig-like domain [General function prediction only].(96.8/7.30e-02)	minor capsid protein (Ig-like domain-containing)
37	23687	24274	+	tail tubular protein A [*Pseudomonas* phage phiIBB-PF7A]<br><br>tail tubular protein A [*Pseudomonas* phage BIM BV-46]<br><br>tail tubular protein A [*Pseudomonas* phage Phi-S1]	6.0e-142<br><br>2e-136<br><br>4e-136	PHA00428 (7-195)<br><br>Tube (14-180)	4.35e-91<br><br>4.60e-60	PF17212.5; Tube ; Tail tubular protein(100.0/2.40e-36)<br><br>PF11650.10; P22_Tail-4 ; P22 tail accessory factor(97.27/1.50e-02)	tail tubular protein A
38	24285	26708	+	tail tubular protein B [*Pseudomonas* phage	0.0e+00			PF11134.10; Phage_stabilise ; Phage stabilisation	tail tubular protein B

				phiIBB-PF7A]<br><br>tail tubular protein B [*Pseudomonas* phage Phi-S1]	 <br>0.0e+00			protein(98.13/2.30e-04)	(stabilisation protein)
39	26737	27174	+	internal virion protein A [*Pseudomonas* phage phiIBB-PF7A]<br><br>internal virion protein A [*Pseudomonas* phage 22PfluR64PP]<br><br>internal virion protein A [*Pseudomonas* phage PFP1]	6.0e-106<br><br>8.0e-93<br><br>1.0e-92	PHA00432 (1-145)<br><br>DUF2833 (46-134)	5.18e-20<br><br>3.36e-13	PF11090.10; ; DUF2833 ; Protein of unknown function (DUF2833)(99.55/2.80e-13)<br><br>d2ge3a1; d.108.1.1 (A:6-169) Probable acetyltransferase Atu2290 {Agrobacterium tumefaciens [TaxId: 358]}(99.55/5.40e-13)<br><br>d1yr0a1; d.108.1.1 (A:4-166) Phosphinothricin acetyltransferase {Agrobacterium tumefaciens [TaxId: 358]}(99.54/6.30e-13)	internal virion protein A
40	27186	27743	+	internal virion protein B [*Pseudomonas* phage phiIBB-PF7A]<br><br>internal virion protein B [*Pseudomonas* phage BIM BV-46]	3.0e-127<br><br>2.0e-126	PHA00101 (1-185)	4.11e-43	('-', '-')	internal virion protein B
41	27752	29965	+	internal virion protein C [*Pseudomonas* phage BIM BV-46]<br><br>internal virion protein C [*Pseudomonas* phage phiIBB-PF7A]	0.0e+00<br><br>0.0e+00	PHA00431 (1-737)	0e+00	('-', '-')	internal virion protein C

42	2996<br>9	3396<br>4	+	internal virion protein D [*Pseudomonas* phage BIM BV-46]<br><br>DNA translocation protein [*Pseudomonas* phage Phi-S1]	0.0e+00<br><br>0.0e+00	PHA00368<br>(11-1331)<br><br>SLT<br>(21-118)	0e+00<br><br>1.14e-19	d1qsaa2; d.2.1.6 (A:451-618) 70 kDa soluble lytic transglycosylase, SLT70 {Escherichia coli [TaxId: 562]}(98.68/1.70e-07)<br><br>COG0741; MltE; Soluble lytic murein transglycosylase and related regulatory proteins (some contain LysM/invasin domains) [Cell wa(98.52/8.70e-07)<br><br>COG2951; MltB; Membrane-bound lytic murein transglycosylase B [Cell wall/membrane/envelope biogenesis].(98.33/2.00e-06)	internal virion protein D
43	3402<br>8	3568<br>0	+	tail fiber protein [*Pseudomonas* phage phiIBB-PF7A]<br><br>tail fiber protein [*Pseudomonas* phage Phi-S1]	0.0e+00<br><br>9.0e-131	PHA00430<br>(1-311)<br><br>Phage_T7_tail<br>(1-156)	1.81e-104<br><br>3.22e-52	PF03906.16; Phage_T7_tail ; Phage T7 tail fibre protein(97.08/4.50e-02)	tail fibre protein
44	3569<br>0	3589<br>3	+	lysis protein [*Pseudomonas* phage phiIBB-PF7A]<br><br>type II holin [*Pseudomonas* phage PFP1]<br><br>type II holin [*Pseudomonas* phage Phi-S1]	2.0e-41<br><br>7.0e-39<br><br>2.0e-37	PHA00426<br>(4-63)	1.29e-13	PF10746.11; Phage_holin_2_2 ; Phage holin T7 family, holin superfamily II(99.86/1.50e-21)<br><br>PF16080.7; Phage_holin_2_3 ; Bacteriophage holin family HP1(96.55/4.10e-02)<br><br>PF16082.7; Phage_holin_2_4 ;	type II holin

								Bacteriophage holin family, superfamily II-like(96.44/5.70e-02)	
45	35886	36143	+	DNA packaging/maturation protein A [*Pseudomonas* phage phiIBB-PF7A]<br><br>DNA packaging protein [*Pseudomonas* phage 22PfluR64PP]<br><br>DNA packaging protein A [*Pseudomonas* phage UNO-SLW1]<br>terminase small subunit [*Pseudomonas* phage Stalingrad]	3.0e-54<br><br>5.0e-53<br><br>1.0e-34<br><br>3.0e-25	PHA00425 (5-78)<br><br>DNA_Packaging_2 (6-73)	2.30e-21<br><br>2.11e-13	PF11123.10; DNA_Packaging_2 ; DNA packaging protein(99.89/1.60e-22)	small subunit terminase
46	36143	36580	+	putative cell lysis protein - endopeptidase [*Pseudomonas* phage phiIBB-PF7A]<br><br>Rz-like lysis protein [*Pseudomonas* phage Phi-S1]	6.0e-99<br><br>3.0e-86	PHA00276 (6-142)	4.66e-16	PF03245.15; Phage_lysis ; Bacteriophage Rz lysis protein(99.74/3.20e-16)<br><br>PF10721.11; DUF2514 ; Protein of unknown function (DUF2514)(98.9/1.10e-07)	Rz-like lysis protein
47	36577	38346	+	DNA packaging protein B [*Pseudomonas* phage phiIBB-PF7A]<br><br>terminase large subunit [*Pseudomonas* phage Stalingrad]	0.0e+00<br><br>0.0e+00			COG5323; COG5323; Large terminase phage packaging protein [Mobilome: prophages, transposons].(100.0/2.10e-30)<br><br>PF03354.17; Terminase_1 ; Phage Terminase(99.98/6.00e-30)	large subunit terminase

								COG4373; COG4373; Mu-like prophage FluMu protein gp28 [Mobilome: prophages, transposons].(99.97/1.80e-28)	
48	38564	38737	+	Phage protein [Yersinia phage fPS-9]<br><br>Phage protein [Yersinia phage fPS-59]<br><br>gene 19.5 [Escherichia phage T7]	1.0e-06<br><br>6.0e-06<br><br>2.0e-05	PHA00406 (12-55)	3.20e-06	PF17553.4; DUF5465 ; Family of unknown function (DUF5465)(97.98/5.60e-06)	DUF5465 domain-containing protein
49	38781	39167	+	hp				('-', '-')	hp

Table S3. Primers used to locate the ends of the Pf-10 genome.

Purpose	Primer name	Primer sequence (5’-3’)	№PCR	Template
Determination of the left genome end	Pf-10_L_rev1	GGATCGCTTTGTCAACCCCT	I PCR	genomic DNA of Pf-10 after DNA tailing reaction
	AncherdT16V	GACCACGCGTATCGATGTCGACTTTTTTTTTTTTTTTTV		
	Pf-10_L_rev2	TGATGGTCCTCTATGGGCCT	II PCR	I PCR product
	Anch	GACCACGCGTATCGATGTCGAC		
Determination of the right genome end	Pf-10_R_for1	CCATGAGCATTGACTTACAGTACA	I PCR	genomic DNA of Pf-10 after DNA tailing reaction
	AncherdT16V	GACCACGCGTATCGATGTCGACTTTTTTTTTTTTTTTTV		
	Pf-10_R_for2	CCTTTAGGGTGCAGCACATC	II PCR	I PCR product
	Anch	GACCACGCGTATCGATGTCGAC		
Additional test. Determination of the right genome end	Pf-10-t	TGAGAATCATGTGCTATCTG	-	Genomic DNA of Pf-10

Table S4. Large terminase subunit of Pf-10 and large terminase subunits of phages with well-known packaging mechanisms used for the phylogenetic tree

Terminus type		Phage	Number GenBank (terminase large subunit)	Sourse
Exact direct terminal repeats	Short direct terminal repeats (T7)	Pseudomonas phage Pf-10	YP_009145642.1	-
		Enterobacteria phage T7	QRE00040.1	[Casjens,2009]
		Enterobacteria phage T3	YP_009792972.1	[Pajunen,2002]
		Yersinia phage phiYeO3-12	NP_052122.1	[Pajunen,2000]
		Pseudomonas phage phi15	YP_004286227.1	[Cornelissen,2011]
		Pseudomonas phage PFP1	YP_009804025.1	[Li,2018]
	Short direct terminal repeats (N4)	Escherichia phage N4	YP_950546.1	[Wittmann,2020]
		Achromobacter phage JWDelta	AHC56597.1	[Ma,2016]
		Erwinia phage vB_EamP-S6	YP_007005834.1	[Born,2011]
	Short direct terminal repeats (c-st)	Clostridium phage c-st	YP_398598.1	[Sakaguchi,2005]
		Bacillus phage Izhevsk	QIW89903.1	[Skorynina,2020]
		Bacillus phage Basilisk	AGR46580.1	[Grose,2014]
	Long DTRs (SPO1)	Bacillus virus SPO1	YP_002300330.1	[Stewart,2009]
		Listeria phage A511	YP_001468454.1	[Klumpp,2008]
		Brochothrix phage A9	YP_004301396.1	[Kilcher,2010]
	Long DTRs (T5)	Enterobacteria phage T5	YP_006983.1	[Casjens,2009]
		Salmonella virus SPC35	YP_004306624.1	[Kim,2011]
		Providencia phage vB_PreS_PR1	YP_009599184.1	[Oliveira,2017]
Cohesive ends	5'cos ends (lambda)	Bacteriophage N15	NP_046897.1	[Ravin,2011]
		Enterobacteria phage lambda	NP_040581.1	[Casjens,2009]
	5'cos ends (P2)	Escherichia virus P2	NP_046758.1	[Casjens,2009], [Christie,2016]
		Escherichia virus 186	NP_052251.1	[Bullas,1991]
		Pseudomonas phage phiCTX	NP_490600.1	[Nakayama,1999]
	3'cos ends (HK97)	Escherichia virus HK97	NP_037698.1	[Juhala,2000]
		Escherichia virus HK022	NP_037663.1	[Juhala,2000]
		RHizobium phage 16-3	YP_002117560.1	[Ganyu,2005]
Host DNA at termini	Host ends (Mu)	Escherichia virus Mu	AAF01106.1	[Morgan,2002]
		Pseudomonas phage B3	YP_164067.1	[Braid,2004]
		Burkholderia virus BcepMu	YP_024701.1	[Summer,2004]
	Host ends (D3112)	Pseudomonas virus MP22	YP_001469154.1	[Heo,2007]
		Haemophilus phage SuMu	YP_007002934.1	[Zehr,2012]
		Pseudomonas virus D3112	NP_938233.1	[Heo,2007]

**Circularly permuted direct terminal repeats**	Headful (P22)	Salmonella virus P22	YP_063734.1	[Byl,2000]
		Salmonella phage ST64T	NP_720326.1	[Mmolawa,2003]
		Enterobacteria phage LP7	AAA88220.1	[Petri,1990]
	Headful (Sf6)	Shigella phage Sf6	NP_958178.1	[Zhao,2003]
		Hamiltonella virus APSE1	NP_050979.1	[van der Wilk, 1999]
		Enterobacteria phage CUS-3	ABQ88401.1	[King,2007]
	Headful (933W)	Enterobacteria phage 933W	NP_049511.1	[Plunkett III,1999]
		Burkholderia virus Bcep22	NP_944278.1	[Gill,2011]
	Headful (phiKZ)	Pseudomonas phage phiKZ	NP_803591.1	[Lecoutere,2009]
		Pseudomonas phage 201phi2-1	YP_001956731.1	[Thomas,2008]
		Erwinia phage phiEaH2	YP_007237828.1	[Dömötör,2012]
	Headful (T4)	Enterobacteria phage T4	NP_049776.1	[Miller,2003]
		Vibrio phage KVP40	NP_899601.1	[Miller,2003]
		Enterobacteria phage RB49	NP_891724.1	[Desplats,2002]
	Headful (phiPLPE)	Yersinia phage PY100	CAJ28416.1	[Schwudke,2008]
		Klebsiella phage JD001	YP_007392855.1	[Cui,2012]
		Iodobacterteriophage phiPLPE	YP_002128452.1	[Leblanc,2009]
	Headful (SPP1)	Bacillus phage vB_BcM_Sam46	QIQ61203.1	[Kazantseva,2021]
		Bacillus phage SPP1	NP_690654.1	[Alonso,1997]
		Staphylococcus virus CNPH82	YP_950600.1	[Daniel,2007]
	Headful (B83)	Bacillus Phage vB_BtS_B83	QCQ57785.1	[Piligrimova,2019]
		Bacillus phage vB_BtS_BMBtp14	YP_009830709.1	[Piligrimova,2019]
**Covalent terminal protein (phi29)**	Protein-primed mechanism [Longás,2008]	Bacillus phage phi29	YP_002004545.1	[Simpson,2000], [Morais,2008], [Longás,2008]
		Bacillus phage Nf	YP_009910733.1	[Longás,2008]

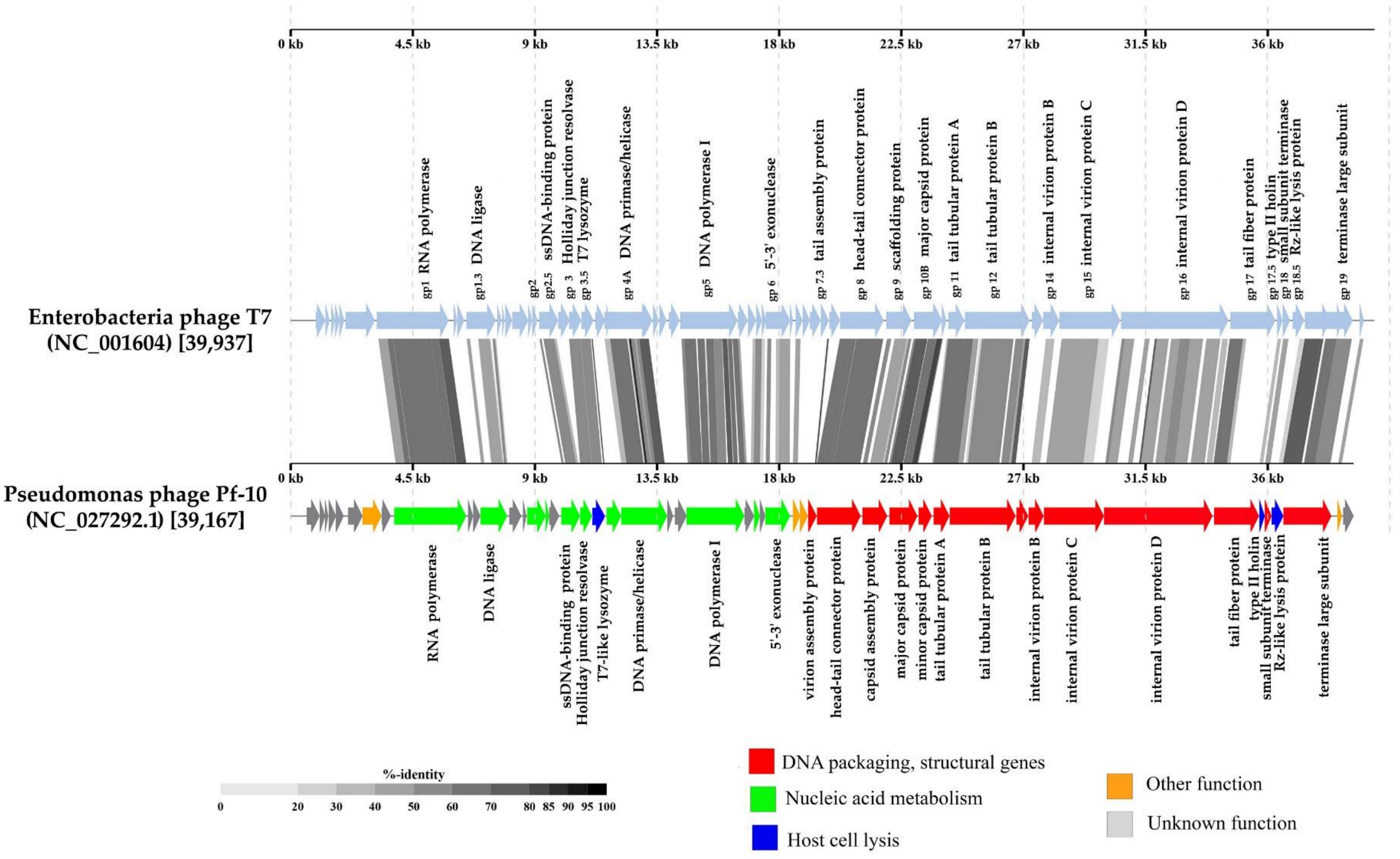

Figure S3. The pairwise whole-genome tBLASTx comparison of *Pseudomonas* phage Pf-10 and *Enterobacteria* phage T7 visualized with ViPTree server version 1.9. The Pf-10 genome color scheme corresponds to Figure 6 in main text (see the legend). Gray areas between the genome maps indicate the level of identity.

Table S5. Phage genomes used for phylogenetic inference.

№	Name	Genome Accession number	Genome length	GC-content, %	ORFs	Number of tRNAs/tmRNAs	BLASTN nucleotide identity to Pf-10, %*	Proteins shared with Pf-10**	
								number	%
1	*Pseudomonas* phage Pf-10	NC_027292.1	39,167	56.5	49	0/0	-	-	-
2	*Pseudomonas* phage BIM BV-46	MT094431.1	38,860	56,4	47	0/0	93.5	43	89.6
3	*Pseudomonas* phage Phi-S1	NC_021062.1	40,192	56.2	51	0/1	89.2	43	86.0
4	*Pseudomonas* phage phiIBB-PF7A	NC_015264.1	40,973	56.3	54	0/0	84.8	42	83.5
5	*Pseudomonas* phage PFP1	NC_047997.1	40,914	55,8	52	0/0	67.9	39	77.2
6	*Pseudomonas* phage 22PfluR64PP	NC_047965.1	40,822	55.8	53	0/0	69.1	39	76.5
7	*Pseudomonas* phage 67PfluR64PP	MH179478.2	40,748	55.9	53	0/0	69.1	39	76.5
8	*Pseudomonas* phage 71PfluR64PP	MH179475.2	40,582	55.9	53	0/0	69.0	39	76.5
9	*Pseudomonas* phage UNO-SLW1	NC_047873.1	39,215	57,9	48	0/0	64.7	34	70.1
10	*Pseudomonas* phage PPpW-4	NC_023005.1	41,386	56.8	52	0/0	53.0	34	67.3
11	*Pseudomonas* phage phi15	NC_015208.1	39,562	58.2	49	0/0	57.7	34	69.4
12	*Pseudomonas* phage Stalingrad	MT711887.1	40,471	57.9	52	0/0	49.7	32	63.4
13	*Pseudomonas* phage Henninger	NC_047922.1	40,923	57.6	53	0/0	38.7	34	66.7
14	*Pseudomonas* phage MR2	MT104466.1	40,989	57.6	52	0/0	37.0	34	67.3
15	*Pseudomonas* phage PPPL-1	NC_028661.1	41,149	57.0	53	0/0	38.5	33	64.7
16	*Pseudomonas* phage MR1	MT104465.1	39,033	56.2	46	0/0	37.7	30	63.2
17	*Pseudomonas* phage phiPSA2	NC_024362.1	40,472	57.4	51	0/0	37.2	34	68
18	*Pseudomonas* phage gh-1	NC_004665.1	37,359	57.4	45	0/0	36.6	31	66.0
19	*Cronobacter* phage Dev2	NC_023558.1	38,966	52.6	48	0/0	18.9	25	51.5
20	*Citrobacter* phage SH4	NC_031018.1	39,274	52.6	50	0/0	19.7	25	50.5
21	*Citrobacter* phage SH3	NC_031123.1	39,444	50.6	49	0/0	17.2	25	51
22	*Escherichia* phage vB_EcoP_GA2A	NC_031943.1	40,470	51.1	54	0/0	17.2	24	46.6
23	*Citrobacter* phage CR8	NC_023548.1	39,651	49.7	52	0/0	19.7	25	49.5
24	*Vibrio* phage N4	NC_013651.1	38,497	42.8	49	0/0	13.2	23	47.0
25	*Vibrio* phage VP3	JQ780163.1	39,481	42.6	54	0/0	12.5	23	44.6
26	*Vibrio* phage VP4	NC_007149.1	39,503	42,6	56	0/0	12.5	23	43.8
27	*Vibrio* phage ICP3	NC_015159.1	39,162	42.9	50	0/0	13.8	23	46.5
28	*Vibrio* phage ICP3_2007_A	HQ641344.1	39,088	42.9	49	0/0	13.8	23	47.0

29	*Vibrio* phage ICP3_2008_A	HQ641343.1	39,349	42.8	51	0/0	13.8	23	46.0
30	*Vibrio* phage ICP3_2009_B	HQ641341.1	39,042	42.8	51	0/0	13.8	22	44.0

*Determined using BLASTn compared to Pf-10 (multiplying % coverage by % identity); **Determined using GET_HOMOLOGUES (COGtriangles algorithm, -G -t 0 -C 75).

Ten phage genomes found by the BLASTn search using the whole Pf-10 genome sequence as the query are light gray.

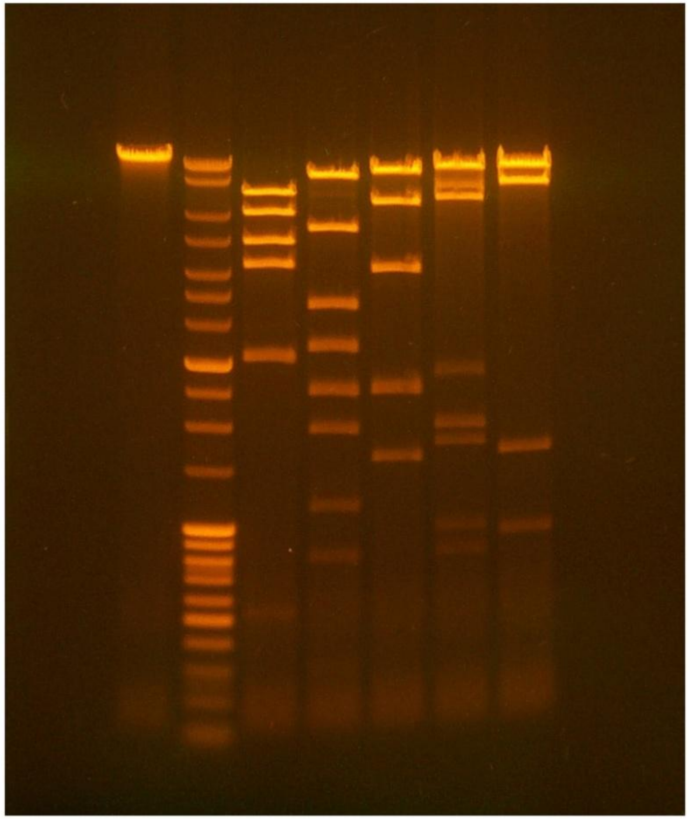

Figure S4. Restriction analysis of the phage genomic DNA. This is the original gel image used to generate Figure 8, A in the main text. Kodak EDAS 290 Gel Documentation System ("Kodak") was used to capture the image. 1 – intact phage DNA; 2 – molecular weight markers; 3 – HindIII; 4 – AfeI; 5 – NdeI; 6 – EcoRI; 7 – EcoRV.

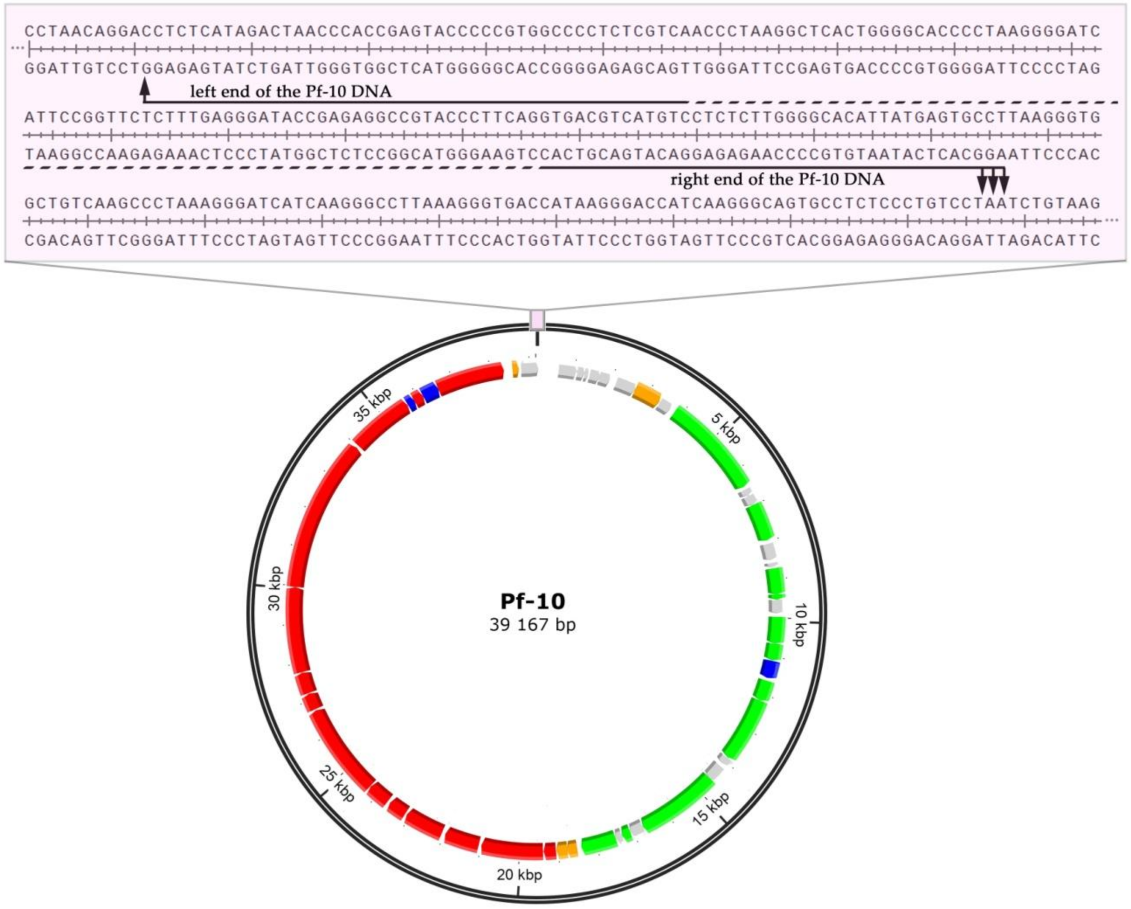

Figure S5. Schematic representation of the DTR-containing region of the Pf-10 genome after whole genome sequencing and genome assembly. The terminal regions of the PCR product sequences obtained with RAGE for right and left ends of the Pf-10 DNA are shown on the sequence. The tentative positions of the Pf-10 genome ends, identified by RAGE method, are indicated with black arrows. The Pf-10 genome color scheme corresponds to Figure 6 in main text (see the legend).
